# The Diesel Tree *Sindora glabra* Genome Provides Insights Into the Evolution of Oleoresin Biosynthesis

**DOI:** 10.3389/fpls.2021.794830

**Published:** 2022-01-04

**Authors:** Niu Yu, Haixi Sun, Jinchang Yang, Rongsheng Li

**Affiliations:** ^1^State Key Laboratory of Tree Genetics and Breeding, Research Institute of Tropical Forestry, Chinese Academy of Forestry, Guangzhou, China; ^2^College of Life Sciences, University of Chinese Academy of Sciences, Beijing, China

**Keywords:** genome evolution, oleoresin, terpene biosynthesis, plant defense, *Sindora glabra*

## Abstract

*Sindora glabra* is an economically important tree that produces abundant oleoresin in the trunk. Here, we present a high-quality chromosome-scale assembly of *S. glabra* genome by combining Illumina HiSeq, Pacific Biosciences sequencing, and Hi-C technologies. The size of *S. glabra* genome was 1.11 Gb, with a contig N50 of 1.27 Mb and 31,944 predicted genes. This is the first sequenced genome of the subfamily Caesalpinioideae. As a sister taxon to Papilionoideae, *S. glabra* underwent an ancient genome triplication shared by core eudicots and further whole-genome duplication shared by early-legume in the last 73.3 million years. *S. glabra* harbors specific genes and expanded genes largely involved in stress responses and biosynthesis of secondary metabolites. Moreover, 59 terpene backbone biosynthesis genes and 64 terpene synthase genes were identified, which together with co-expressed transcription factors could contribute to the diversity and specificity of terpene compounds and high terpene content in *S. glabra* stem. In addition, 63 disease resistance NBS-LRR genes were found to be unique in *S. glabra* genome and their expression levels were correlated with the accumulation of terpene profiles, suggesting potential defense function of terpenes in *S. glabra.* These together provide new resources for understanding genome evolution and oleoresin production.

## Introduction

*Sindora glabra*, also known as diesel tree, is a woody important plant that exudes a sesquiterpene-rich oleoresin when the trunk is tapped (Supplementary Figure 1). This oleoresin has great potential for utilization as medicine, essential oil, pesticide and fuel (da Trindade et al., 2018; Yu et al., 2020a). *S. glabra* is natively distributed in Vietnam, Thailand, Malaysia, Philippines, and Hainan Island of China, and now widely cultivated in tropical areas of China including Guangdong, Guangxi, and Fujian provinces. *S. glabra* tree can reach to 20 m height and 2 m diameter at breast height. Their cylindrical trunks contain intercellular secretory canals distributed along the marginal parenchyma bands and arranged in tangential direction, and the oleoresin is stored in parenchyma cells of the canals (Yu et al., 2020b). *S. glabra* belongs to the subfamily Caesalpinoideae of family Fabaceae. This subfamily encompasses about 3,000 species spread across 180 genera of shrubs and trees. Besides *S. glabra*, Caesalpinoideae includes a number of plants with economic importance, such as *Bauhinia variegate*, *Cassia tora* and *Caesalpinia sappan*. In addition, the oleoresin in *S. glabra* has some common features with those in the genus *Copaifera*, which has been widely used as a traditional medicine in Neotropical regions for thousands of years (da Trindade et al., 2018). Although the phylogenetic relationships of legumes have been extensively studied over the past two decades, there are still some debates about the deep relationships among the subfamilies Caesalpinioideae and Papilionoideae (Zhang et al., 2020). Moreover, the sequenced plant genomes in legumes have been primarily restricted to the subfamily Papilionoideae, such as *Glycine max* (Schmutz et al., 2010), *Medicago truncatula* (Young et al., 2011), *Arachis duranensis* and *Arachis ipaensis* (Bertioli et al., 2016), and also the subfamily Mimosaceae, such as *Mimosa pudica* (Griesmann et al., 2018). Therefore, the uncovering of *S. glabra* genome could provide useful resources for deciphering the genome evolution.

Terpenoids are the largest and structurally most diverse group of plant secondary metabolites derived from natural sources. They play important physiological and ecological roles that include hormones, signals, defense and stress response, etc. (Karunanithi and Zerbe, 2019). Although there are a few hundred terpenes that are found in almost all plants such as primary metabolites, the majority of terpenes are produced and stored in a specific lineage or even a single species (Pichersky and Raguso, 2018). The oleoresin in *S. glabra* are mainly composed of sesquiterpenes, among which α-copaene and β-caryophyllene accounted for over 50% of total sesquiterpenes (Yu et al., 2018), whereas gymnosperm oleoresin are almost universally composed of mono- and diterpenes (Karanikas et al., 2010). In addition, the trunk of *S. glabra* could produce a large volume of sesquiterpenes, which are significantly higher than the trace amount of terpenes in other plant tissues such as trichomes, flowers, leaves, and roots (Courtois et al., 2012; Wittayalai et al., 2014; Aizpurua-Olaizola et al., 2016; Livingston et al., 2020). Investigating the mechanisms of unique oleoresin biosynthesis in *S. glabra* may provide new insights into the evolution of terpene production.

Terpenes are important components of the plant stress overcoming mechanism. They are dedicated to mediating cooperative interactions with other organisms or environmental defense and adaptation (Agrawal and Heil, 2012; Tholl, 2015). In conifer trees, terpenes exhibit potent toxicity and serve pivotal functions in chemical defenses against herbivores, insect pests, and microbial pathogens (Miller et al., 2005; Schmelz et al., 2014). Spruce tree with pathogen-induced higher terpene levels was less susceptible to bark beetle attack (Mageroy et al., 2019). Environmental stress such as drought, temperatures, light, and salinity greatly affected the production and accumulation of terpenes (Sallas et al., 2003; Turtola et al., 2003). However, the mechanisms of how terpene modulates the responses to abiotic stressors are still unclear. *S. glabra* is the dominant upper standing tree in the monsoon rain forest. It has strong adaptability and drought tolerance, and seldom suffers from disease except for occasional leaf rust disease. We hypothesized that the copious volume of terpene oleoresin in *S. glabra* plays important defensive functions in order to cope with various biotic and abiotic stresses. Nevertheless, deciphering the putative functions has been hampered by a lack of genetic and genomic information. In addition, previous studies on oleoresin biosynthesis in *S. glabra* was restricted to limited genes identified by the transcriptomes (Yu et al., 2018, 2020a), genome-wide identification of terpene biosynthesis genes are necessary for comprehensive understanding of this complex biological process.

In this study, we report the first high-quality assembly of *S. glabra* genome using a combined strategy. Comparative genomic analysis was performed to investigate the evolutionary relationship among different plant species. Comprehensive analysis of genes involved in terpene biosynthesis and environmental adaptability was conducted to elucidate the genetic basis of terpene biosynthesis and potential biological functions of terpene.

## Materials and Methods

### Genomic Sequencing

The individual plant of *S. glabra* used for genome sequencing was originally collected from Bawangling (19°01′41′′N, 109°07′11′′E), Changjiang City, Hainan Island in Southern China and cultivated in the Research Institute of Tropical Forestry. Genomic DNA was extracted from young leaves of *S. glabra* plants using the DNeasy Plant Mini Kit (Qiagen, Germany) according to the protocol and then subjected to DNA library preparation using established Illumina pair-end protocols. Genome size was initially estimated through flow cytometry analysis (Galbraith et al., 1983). Leaves from the sequenced *S. glabra* seedling were collected and subject to analysis using the Partec CyStain UV Precise P kit on the Partec CyFlow Space (Sysmex Partec, Görlitz, Germany). *Jatropha curcas* with a genome size 416 Mb was used as standard reference (Carvalho et al., 2008). Estimation of genome size based on Illumina Hiseq2000 short reads was conducted via a 17 bp *k*-mer frequency as implemented in SOAPdenovo2 (Luo et al., 2015). Heterozygous rate was determined by the *k*-mer distribution and GenomeScope (Vurture et al., 2017). DNA library was sequenced using an Illumina Hiseq 2000 platform (Illumina, CA, United States). For PacBio long-read sequencing, a 20-kb single-molecule real-time (SMRT) DNA sequencing library was constructed with a Template Prep Kit (Pacific Biosciences, CA, United States) and SMRT cells were run on the PacBio Sequel II system with P6-C4 chemistry.

### *De novo* Genome Assembly

PacBio subreads were used for preliminary assembly with Falcon (Chin et al., 2016) to generate the contig. Primary contigs were polished using Quiver (Chin et al., 2013). Paired-end clean reads from Illumina platform were aligned to the assembly using BWA (Li et al., 2010). Base-pair correction of the assembly was performed using Pilon (Walker et al., 2014). Pilon mostly corrected single insertions and deletions in regions enriched with homopolymer. Contigs or scaffolds shorter than 10 kb were excluded from the overall analysis to avoid results from spurious misassembly. The Hi-C library was prepared using ligation protocols (Belaghzal et al., 2017). Raw Hi-C data were generated using Illumina HiSeq PE150 sequencing platform. After filtering low-quality reads, clean reads were firstly mapped to the assembled scaffolds using BWA software (Li and Durbin, 2010). The high quality paired-end Hi-C reads were then filtered using HiCUP (Wingett et al., 2015). The scaffolds were clustered, ordered, and orientated onto the chromosomes using the valid read pairs by LACHESIS (Burton et al., 2013). The integrity and accuracy of the final assembly was evaluated using BUSCO (Simão et al., 2015) and CEGMA analysis (Parra et al., 2007), and further assessed by mapping RNA-seq data to the assembled genome.

### Genome Annotation

Genome annotation was conducted using a combined strategy based on *ab initio* prediction, homology alignment, and RNA-seq assisted prediction. Repeat annotation was firstly built *ab initio* using repetitive elements database by LTR_FINDER, RepeatScout, RepeatModeler with default parameters (Xu and Wang, 2007; Flynn et al., 2020). Then all repeat sequences with lengths >100 bp and gap ‘N’ less than 5% constituted the raw transposable element (TE) library. A combination of Repbase database and *ab initio* TE library was supplied to RepeatMasker software with default parameters for DNA-level repeat identification (Jurka et al., 2005; Smit et al., 2010).

To annotate gene structural models, homologous protein sequences were aligned to the genome using TblastN (v2.2.26; E-value ≤ 1e-5) (Altschul et al., 1990), and then the matching proteins were aligned to the homologous genome sequences for accurate spliced alignments with GeneWise (v2.4.1) software (Birney et al., 2004), which was used to predict gene structure contained in each protein-coding region.

To optimize the genome annotation, the leaf, stem, and root tissues from 2-year-old *S. glabra* seedlings, and adult stems from a 25-year-old tree were harvested and subject to RNA sequencing using Illumina HiSeq 2500 platform. The RNA-seq reads from different tissues were then aligned to the assembled genome using TopHat (v2.0.11) with default parameters to identify exons region and splice positions (Kim et al., 2013). The alignment was then used as input for Cufflinks (v2.2.1) with default parameters for genome-based transcript assembly (Trapnell et al., 2010). Unigenes were aligned to the assembled genome using PASA (Program to Assemble Spliced Alignment) to annotate protein-coding genes and alternatively spliced isoforms (Haas et al., 2003). Then all predications were combined with EvidenceModeler (EVM v1.1.1) (Haas et al., 2008) to produce a consensus gene set. Finally, the non-redundant reference gene set was generated by removing homologous transposons.

Gene functions were assigned according to the best match by aligning the protein sequences to the Swiss-Prot using Blastp (with a threshold of *E*-value ≤ 1e-5) (Bairoch and Apweiler, 2000). The motifs and domains were annotated using InterProScan70 (v5.31) by searching against publicly available databases, including ProDom, PRINTS, Pfam, SMRT, PANTHER and PROSITE (Mulder and Apweiler, 2007). The Gene Ontology (GO) IDs for each gene were assigned according to the corresponding InterPro entry (Ashburner et al., 2000). Proteins function was predicted by transferring annotations from the closest BLAST hit (*E*-value < 10^–5^) in the Swissprot20 database and DIAMOND (v0.8.22)/BLAST hit (*E*-value < 10^–5^) in the NR database. Gene set was mapped to KEGG pathway and the best match for each gene was then identified (Kanehisa and Goto, 2000).

The tRNAs were predicted using the program tRNAscan-SE (Lowe and Eddy, 1997) with eukaryote parameters. The rRNAs were identified by RNAmmer (Lagesen et al., 2007). The miRNAs, snRNAs were identified by searching against the Rfam database with default parameters using the infernal software (Nawrocki and Eddy, 2013).

### Comparative Genome Analysis

Orthologous relationships between genes of 16 species (*G. max*, *Vigna unguiculata*, *Phaseolus vulgaris*, *Spatholobus suberectus*, *Cicer arietinum*, *M. truncatula*, *Cinnamomum micranthum*, *Betula pendula*, *Eucalyptus grandis*, *Olea europaea*, *Theobroma cacao*, *Arabidopsis thaliana*, *Populus trichocarpa*, *Hevea brasiliensis*, and *Oryza sativa*) were inferred through all-against-all protein sequence similarity searches with OthoMCL (Li et al., 2003). *S. glabra* specific genes were obtained from the clustering of gene families from four Leguminosae plant genomes (*S. glabra, S. suberectus, V. unguiculata*, and *P. vulgaris*). For each gene family, an alignment was produced using Muscle (Robert, 2004) and the ambiguously aligned positions were trimmed using Gblocks (Talavera and Castresana, 2007). Phylogenetic tree was inferred using maximum likelihood in RAxML 7.2.9 (Stamatakis, 2014) with monocotyledon *O. sativa* as the out-group. Divergence times between species were calculated using MCMC tree program implemented in the PAML (Yang, 2007). The TimeTree database was used to calibrate the divergence time^1^.

In order to identify gene family evolution as a stochastic birth and death process and where gene family either expands or contracts per gene per million years independently along each branch of the phylogenetic tree, we used the likelihood model originally implemented in the software package CAFÉ (De Bie et al., 2006). The phylogenetic tree topology and branch lengths were taken into account to infer the significance of changes in gene family size in each branch. Genes that belong to expanded and contracted gene families were subject to GO and KEGG enrichment analysis.

Positive selection pressure was calculated using the branch-site model with PAML. MCScanX was used for detection of syntenic regions with the all-to-all BLASTP results and synteny plot was drawn with dot plotter in MCScanX package (Tang et al., 2008). The 4DTV was calculated using KaKs_calculator (Zhang et al., 2006) with the NG model and whole genome duplication events were identified by 4DTv analysis of gene pairs detected by MCscan.

Blast and hidden Markov models search (HMMsearch) methods were used to identify *TPS* and *NBS-LRR* gene families in the *S. glabra* genome with an *E*-value cut-off of 1.0. TPS gene family was identified by using conserved domain Pfam ID PF01397 or PF03936 as query. All identified TPSs contain a conserved domain structure with the PF01397 or PF03936 and many of TPSs contain both of these domains. The amino acid sequence of the NB-ARC domain Pfam ID PF00931 was used as a query to search for NBS-LRR proteins in *S. glabra* genome.

## Results

### Genome Sequencing and Assembly

The genome of *S. glabra* was sequenced by integrating Illumina sequencing, PacBio single molecular long-read sequencing, and high-throughput chromosome conformation capture (Hi-C) technologies (Supplementary Figure 2). Initially, genome size was estimated by flow cytometry. By comparing the peak fluorescence intensity of *S. glabra* (97.73) to that of *J. curcas* internal standard (33.13) (Carvalho et al., 2008), the genome size of *S. glabra* was estimated to be 1.23 Gb (Supplementary Figure 3A). Then a total of 49.6 Gb raw data were obtained for *k*-mer analysis and the effective rate was 99.9%. According to 17-mer analysis, the estimated genome size of *S. glabra* was 1.22 Gb (Table 1), which was quite close to that estimated by flow cytometry, and the heterozygous rate was estimated at 0.61% (Supplementary Figure 3B). By using PacBio sequencing, 134 Gb of raw data were generated, representing approximately 118-fold coverage of the *S. glabra* genome (Supplementary Figure 4). Based on these 184 Gb (161-fold) data, the draft genome of *S. glabra* was assembled with a final genome size of 1.11 Gb, and a contig N50 size of 1.3 Mb (Supplementary Table 1). The assembled genome contained 1,439 contigs with a maximum length of 5.6 Mb.

**TABLE 1:** Statistics of genome assembly and annotation.

Type	Parameter	Size
Assembly	Estimated genome size	1.22 Gb
	Total assembly size	1.11 Gb
	Sequences anchored to Hi-C map	1.09 Gb
	Number of scaffolds	363
	N50 of scaffolds	84.87 Mb
	Longest scaffolds	136.07 Mb
	Number of Contigs	1,461
	N50 of contigs	1.27 Mb
	Longest contigs	520 Mb
	GC content	28.01%
Annotation	Number of genes	31,944
	Mean transcript length	4,239 bp
	Mean coding sequence length	1,143 bp
	Mean number of exons per gene	5.02
	Mean exon length	227.58 bp
	Mean intron length	769.45 bp
	Number of miRNA	701
	Number of tRNA	672
	Number of rRNA	174
	Number of small nuclear RNA	2,848
	Repeat content	52.40%

**FIGURE 1:**
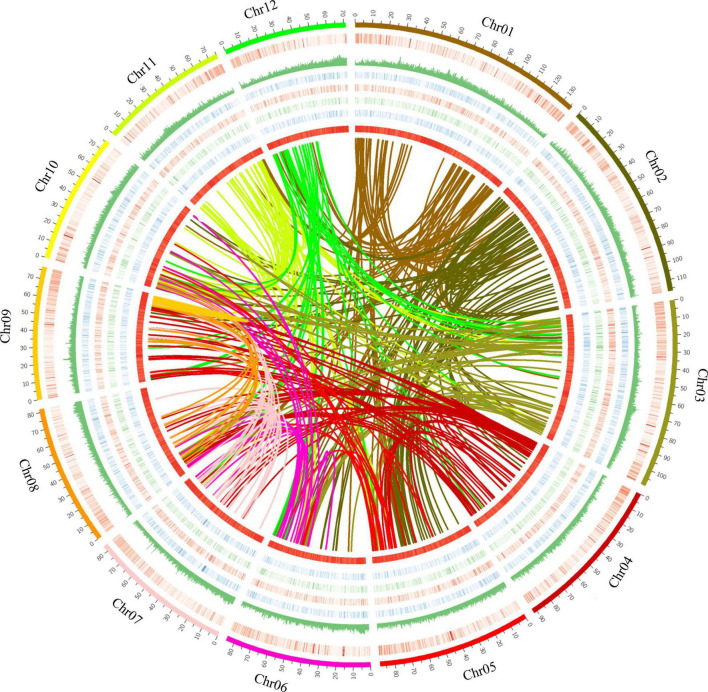
The genomic landscape of *Sindora glabra* tree. The features from outside to inside are chromosomes, gene density (0–1), repeat coverage (0–1), expression levels (0–15) from adult stems, leaf, root, and young stems, GC content (0–0.5), and genome synteny. Twelve chromosomes were ordered by megabases size and featured in 200 kb intervals across the chromosomes. Arcs with different colors indicated syntenic blocks. Circos was used to construct the diagram.

To assemble the scaffolds into pseudochromosomes, Hi-C library was constructed and sequenced, resulting in 137.8 Gb (121-fold) data. After Hi-C data alignment and cluster, a total of 1.09 Gb (98.0% coverage) genome sequences were assembled and anchored onto 12 pseudochromosomes (Figure 1 and Supplementary Figure 5) (Goldblatt, 1981). The length of pseudochromosomes ranged from 71.1 to 136.1 Mb (Supplementary Table 2) and the scaffold N50 was 84.87 Mb (Supplementary Table 3).

Benchmarking Universal Single-Copy Orthologs (BUSCO) evaluation revealed that 1,174 (81.5%), 136 (9.4%), 26 (1.8%), and 105 (7.3%) of BUSCO genes were single-copy, duplicated, fragmented, and missing, respectively (Supplementary Table 4). Core Eukaryotic Genes Mapping Approach (CEGMA) analysis indicated that 95.16% (236) of the CEGs were covered by the assembled genome. The short reads generated from Illumina sequencing were aligned to the assembled genome to check the coverage rate using BWA software. The results showed that 94.1% of the reads could be mapped back to the genome and the coverage rate was 97.6% (Supplementary Table 5). Single Nucleotide Polymorphisms (SNP) calling showed that heterozygosis SNP rate was 0.53% and homology SNP rate was 0.039% (Supplementary Table 6). These data suggested that the *S. glabra* genome assembly is precise and complete and of high quality at the chromosome scale.

### Genome Annotation

Genome annotation was performed by combining the results from *de novo* predictions, homology information from six homologous species including *V. unguiculata*, *S. suberectus*, *P. vulgaris*, *C. arietinum*, *M. truncatula*, *G. max*, and RNA sequencing data. A total of 31,944 genes were predicted, with transcript length of 4,239 bp, coding sequence length of 1,143 bp, exon number of 5.02 per gene, exon length of 227 bp, and intron length of 769 bp on average (Supplementary Table 7). The number of protein-coding genes in *S. glabra* genome was similar to those in *M. truncatula* and *S. suberectus*, and much less than those in *G. max* (Supplementary Table 8). The exon-intron structure of genes showed high conservation between *S. glabra* and *S. suberectus* in terms of transcript and CDS length, exon number, exon and intron length (Supplementary Figure 6).

There were 82.45% (26,338) of annotated genes supported by RNA-seq data (Supplementary Figure 7). Among the predicted 31,944 genes, 29,184 genes (91.4%) were functionally annotated in the six public databases (Supplementary Table 9). NCBI Nr and Interpro blast analyses allowed the annotation of 90.70% and 87.80% of genes, respectively. KEGG analysis annotated 23,660 (74.10%) genes, which is much higher than that in tung tree (24%) (Zhang et al., 2019), but similar to that in rubber tree (72.8%) (Lau et al., 2016). In addition, we predicted 701 miRNAs, 672 tRNAs, 174 rRNAs, and 2848 small nuclear RNAs in the *S. glabra* genome (Supplementary Table 10).

In the *S. glabra* genome, 52.40% of repeat sequences were annotated, including 3.54% of tandem repeat and 51.32% of interspersed repeat. The long terminal repeat (LTR) retrotransposons were the most abundant, accounting for 33.53% of the assembled *S. glabra* genome (Supplementary Figure 8). The two superfamilies of LTR retrotransposons, *Gypsy* and *Copia*, accounted for 11.6 and 12.9% of the assembled genome, respectively (Supplementary File 1). In addition to class I retrotransposons, DNA transposons accounted for 5.00% of the assembled *S. glabra* genome (Supplementary Table 11). Moreover, 15.33% of the *S. glabra* genome comprised unknown repetitive sequences, which is much higher than that in other Papilionoideae species such as *V. unguiculata* (5.7%) (Lonardi et al., 2019) and wild soybean W05 (5.9%) (Xie et al., 2019), but similar to that in *Eucommia ulmoides* (17%) (Wuyun et al., 2018) and *H. brasiliensis* (13%) (Lau et al., 2016).

### Evolution of *S. glabra* Genome

**FIGURE 2:**
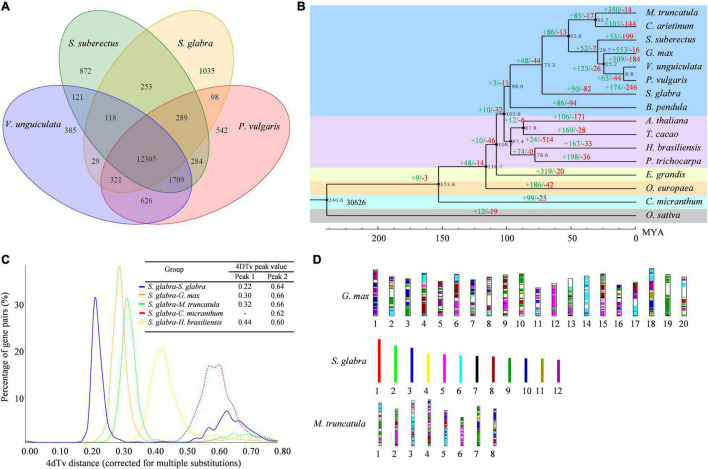
Evolution of *S. glabra* genome. **(A)** A Venn diagram of common and unique gene families among four Leguminosae species based on the gene family cluster analysis. **(B)** Phylogenetic tree of 16 species based on single-copy gene families. The black number at each branch point denotes the estimated divergence time. The value above each branch denotes the number of gene family expansion (in green)/contraction (in red) at each round of genome duplication after divergence from the common ancestor. Bootstrap value for each node is 100. **(C)** Density distribution of 4dTv values. The peak value is shown in the inset. The blue line represents the 4dTv of paralogous genes in *S. glabra*. The orange, green, red and yellow dotted lines represent 4dTv of orthologous gene pairs between *S. glabra* and *G. max*, *S. glabra* and *M. truncatula*, *S. glabra* and *C. micranthum*, *S. glabra*, and *H. brasiliensis*, respectively. **(D)** Schematic representation of syntenic genes among *S. glabra*, *G. max*, and *M. truncatula*. Syntenic blocks were determined by using all 20 chromosomes of *G. max*, 12 chromosomes of *S. glabra* and 8 chromosomes of *M. truncatula.* The same color indicates matched gene pairs.

The protein sequences of 16 species including *G. max*, *V. unguiculata*, *P. vulgaris*, *S. suberectus, C. arietinum*, *M. truncatula*, *C. micranthum*, *B. pendula*, *E. grandis*, *O. europaea*, *T. cacao*, *A. thaliana*, *P. trichocarpa*, *H. brasiliensis*, and *O. sativa* were used for clustering analysis of gene family (Supplementary Table 12). A total of 30,641 gene families were identified from the 16 species, among which 6,751 gene families, including 154 single-copy gene families, were shared (Supplementary Figure 9). Moreover, 12,305 gene families were found to be common in the four species (*S. glabra*, *S. suberectus*, *V. unguiculata*, and *P. vulgaris*) of Fabaceae family (Figure 2A), while 1,035 gene families (1,975 genes) were specific to *S. glabra* (Supplementary File 2). Among these unique genes, 1,740 genes were annotated in five databases (Supplementary Figure 10). Gene Ontology (GO) annotation of the *S. glabra*-unique families revealed significant enrichment of genes involved in the phloem development (GO:0010088) (11 genes), terpene synthase activity (GO:0010333) (10 genes) and signal transduction (GO:0007165) (33 genes) (Supplementary File 3). It was notable that 10 terpene synthase genes were uncovered to be unique in *S. glabra*, suggesting specific functions of these genes in *S. glabra*.

A phylogenetic tree was generated based on single-copy genes from 16 species. The results showed that *S. glabra* belonged to a sister taxon of Papilionoideae subfamily, which was clustered together with *B. pendula* from Betulaceae (Figure 2B). This phylogenetic tree is consistent with the Angiosperm Phylogeny Group (APG) classification (Chase et al., 2016). Moreover, it was estimated that *S. glabra* lineage diverged from the Papilionoideae subfamily approximately 73.3 million years ago (mya), after the divergence of Fagales (98.0 mya).

Expansion and contraction analysis of 30,626 gene families as indicated by the phylogenetic tree, revealed that *S. glabra* expanded 50 gene families (370 genes) and contracted 82 gene families (247 genes) (Figure 2B). Among the expanded gene families, 209 gene families were annotated in the GO database, respectively. GO annotation revealed highly enriched genes related to enzyme inhibitor activity (26 genes), abscisic acid-activated signaling pathway (12 genes), defense response (12 genes), anatomical structure development (11 genes), and small molecule binding (55 genes) (Supplementary File 4). Among the contracted gene families, 225 gene families were annotated in the GO database. GO enrichment analysis showed highly enriched genes involved in nucleotide binding (138 genes), primary metabolic process (108), and catalytic activity (178) (Supplementary File 5). These suggested that the expanded genes in *S. glabra* genome might play important roles in response to stress response and terpene biosynthesis.

When using *S. suberectus*, *V. unguiculata*, *P. vulgaris*, *C. arietinum*, and *M. truncatula* as background, 566 positively selected genes were found in *S. glabra* genome, of which 329 genes were annotated in the GO database. GO enrichment analysis revealed highly enriched genes related to nucleic acid metabolic process (75 genes), cellular aromatic compound metabolic process (81), macromolecule metabolic process (124), and cellular metabolic process (130) (Supplementary File 9).

### Whole Genome Duplication and Collinearity

The fourfold degenerate transversion rate (4DTv) analysis of paralogs and orthologs revealed that *S. glabra* displayed a two-peak pattern for the distribution of 4DTv (Figure 2C), with the left sharp peak corresponding to the early-legume duplication (Schmutz et al., 2010), and the right highly diffuse peak corresponding to the ancient r triplication shared by the core eudicots. The low 4DTv peak value of *S. glabra* indicated that *S. glabra* may have undergone recent whole-genome duplication after the divergence from Papilionoideae family 73.3 mya.

Plotting collinear regions identified 353 syntenic blocks containing 8,665 collinear gene pairs within the *S. glabra* tree genome (Figure 1 and Supplementary File 7). Overall, 12,279 genes comprised the collinear gene pairs, accounting for 38.44% of *S. glabra* genes, a proportion is similar to that in *Morchella esculenta* (33.86%), but considerably higher than that in tung tree (12.52%) (Zhang et al., 2019). In addition, *S. glabra* exhibited strong synteny with *G. max* (51.30%) and *M. truncatula* (49.74%), but weaker synteny with *C. micranthum* (33.71%) and *H. brasiliensis* (32.25%) (Figure 2D).

The *S. glabra* genome shared 1,260 syntenic blocks containing 39,829 collinear gene pairs with *G. max*, and 878 syntenic blocks containing 23,940 collinear gene pairs with *M. truncatula* (Figure 2D). Comparison between *S. glabra* and *G. max* gene blocks revealed that many regions of the *S. glabra* genome have two, but some have three or more, homologous regions in *G. max*. The collinear regions between *S. glabra* and *M. truncatula* generally showed one-to-two synteny relationships. Moreover, significant macrosynteny was found among *S. glabra, G. max*, and *M. truncatula*. Conserved blocks, sometimes as large as chromosome arms, span most euchromatin in all three genomes. For example, chromosome 5 of *S. glabra* corresponded to chromosome 12 of *G. max* and chromosome 6 of *M. truncatula*, chromosome 6 of *S. glabra* corresponded to chromosome 14 of *G. max* and chromosome 3 of *M. truncatula*, and chromosome 9 of *S. glabra* corresponded to chromosome 19 of *G. max* and chromosome 7 of *M. truncatula*. These indicated that these large conserved blocks may be legume specific regions.

### Oleoresin Biosynthesis in *S. glabra*

**FIGURE 3:**
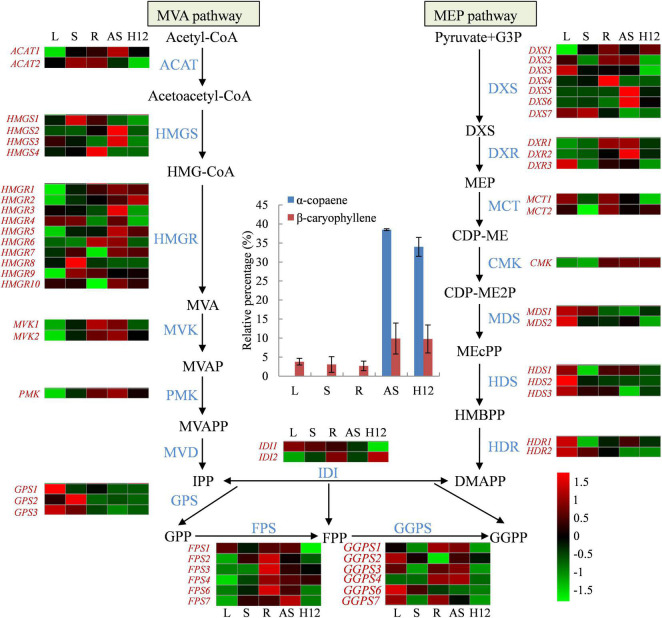
The terpene backbone biosynthesis pathway in *S. glabra*. The terpene backbone biosynthesis is catalyzed by seventeen enzymatic steps with multiple isozymes in each step. The expression levels of genes are presented with the heat map. The bars in the center area exhibit the α-copaene and β-caryophyllene content in five various tissues. L, leaf; S, stem from young plants; R, root; AS, stem from adult plants; H12, stem from high oil-yielding plant. ACAT, acetyl-CoA acetyltransferase; HMGS, hydroxymethylglutaryl -CoA synthase; HMGR, hydroxymethylglutaryl-CoA reductase; MVK, mevalonate kinase; PMK, phosphomevalonate kinase; MVD, mevalonate diphosphate decarboxylase; DXS, 1-deoxy-xylulose 5-phosphate synthase; DXR,1-deoxy-D-xylulose-5-phosphate reductoisomerase; MCT, 2-C-methyl- erythritol 4-phosphate cytidylyltransferase; CMK, 4-(cytidine 5′-diphospho) -2-C-methyl-D-erythritol kinase; MDS, 2-*C*-methyl-D-erythritol 2,4-cyclodiphosphate synthase; HDS, (E)-4-hydroxy-3-methylbut-2-enyl-diphosphate synthase; HDR, 4-hydroxy-3-methylbut-2-enyl diphosphate reductase; IDI, isopentenyl di-phosphate isomerase; GPS, Geranyl diphosphate synthase; FDPS, farnesyl pyrophosphate synthase; GGPS, geranylgeranyl diphosphate synthase.

Oleoresin in *S. glabra* stem is mainly composed of sesquiterpenes (∼85%), among which α-copaene and β-caryophyllene occupied the majority of sesquiterpenes (∼55%) (Yu et al., 2018). To gain insight into the mechanism of terpene biosynthesis, we first measured the content of α-copaene and β-caryophyllene in the same samples that were used for RNA-seq, including leaf (L), stem (S), and root (R) from 2-year-old seedlings, adult stem (AS) from 25-year-old tree, and stem from high oleoresin-producing adult tree H12 (Yu et al., 2018). The compound α-copaene was not observed in all three tissues from the young tree, but α-copaene content accumulated to 39 and 34% in the adult tree and H12 tree, respectively (Figure 3). The β-caryophyllene content also showed higher accumulation in the adult tree. According to the *S. glabra* genome, 59 terpene backbone biosynthesis genes were identified and their expression patterns were further analyzed (Supplementary File 8). Interestingly, the gene family with the largest number in the terpene backbone biosynthesis pathway is *HMGR* (10 genes), which encoded the rate-limiting enzyme in the MVA pathway, indicating important and diverse roles of these genes in *S. glabra* terpene biosynthesis. There were seven genes encoding DXS, FPS, GGPS in the *S. glabra* genome, respectively. Moreover, the genes involved in the MVA pathway exhibited a high level of stem-specific expression. By Pearson correlation analysis between gene expression and terpene accumulation, the expression levels of *HMGR5, HMGR1*, and *HMGR2* were positively correlated with the accumulation rate of α-copaene and β-caryophyllene (Supplementary Figure 11), whereas the expression levels of *IDI1* and *TPS46* were negatively correlated with the accumulation rate of α-copaene and β-caryophyllene in *S. glabra* tree. These results suggested that *HMGR* and *IDI* genes were mainly responsible for the control of sesquiterpene biosynthesis in *S. glabra* tree.

**FIGURE 4:**
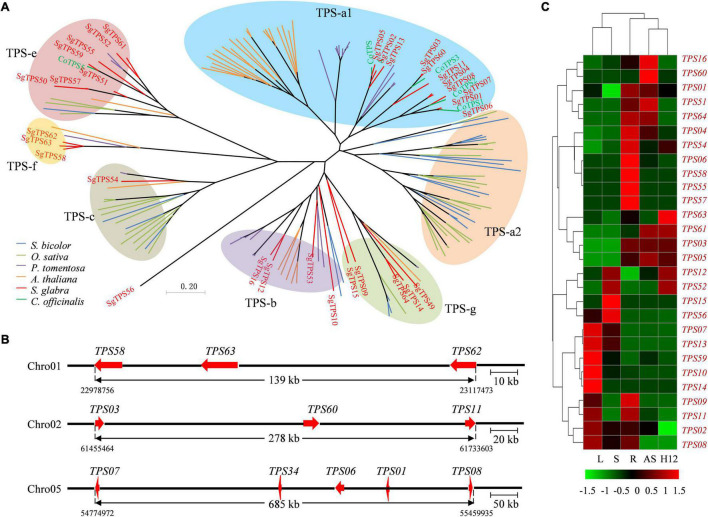
Phylogenetic analyses, gene distribution and expression profiles of terpene synthase genes (TPSs). **(A)** Phylogenetic analysis of putative full-length TPSs from *S. glabra*, *S. bicolor*, *O. sativa*, *P. tomentosa*, *A. thaliana*, and *C. officinalis*. The TPSs from *S. glabra* were indicated in red. **(B)** Distribution of *SgTPS* gene clusters across different chromosomes. Arrows indicate the orientation of genes. **(C)** Heat map of expression patterns of putative full-length *SgTPS* genes. L, leaf; S, stem from young plants; R, root; AS, stem from adult plants; H12, stem from high oil-yielding plant.

To reveal the genes for the specificity and diversity of terpene in *S. glabra*, 64 members of terpene synthase (TPS) gene family were identified in the *S. glabra* genome (Supplementary File 9), which was similar to the number (68) of *TPS* genes in *P. trichocarpa* (Chen et al., 2011). These *TPS* genes were distributed on nine of the 12 chromosomes in *S. glabra* genome (Supplementary Figure 12), being a significant proportion (78.5%) of them located in chromosomes 1, 2, 5, and 9, and none of them in chromosomes 3, 6, and 11. Moreover, 39 (61%) *TPS* genes were organized in five distinct clusters, covering from 5 to 11 genes. The protein length of these *TPS* genes ranged from 55 to 1,156 amino acids (aa), among which 32 TPS genes encoded proteins larger than 500 aa and therefore likely to encode full-length TPS proteins (Chen et al., 2011). Overall amino acid identity among the 32 TPS genes was 34.61%, although it varied greatly from 21% (SgTPS2 and SgTPS59) to 95.26% (SgTPS4 and SgTPS8) (Supplementary File 10). To explore the evolutionary relationship of plant *TPS* genes, 32 putative full-length *SgTPS* genes, and full-length *TPS* genes from *A. thaliana*, *Paulownia tomentosa*, *Sorghum bicolor*, *O. sativa*, and *Calendula officinalis* were used to construct the phylogenetic tree (Yu et al., 2020a). Phylogenetic analyses revealed that 11, 4, 2, 10, and 5 SgTPSs were classified into TPS-a1, b, c, e/f, and g subfamily, respectively (Figure 4A). Moreover, SgTPSs were closely related to CoTPSs from *C. officinalis* rather than from *P. tomentosa* and *A. thaliana*, and quite distant to TPS from monocotyledon *S. bicolor* and *O. sativa*, indicating lineage-specific expansion of *TPS* genes in plant evolution.

Considering the intron/exon number, SgTPS genes were classified into two major classes: I (10–14 introns) and III (6 introns) (Supplementary Figure 13) (Trapp and Croteau, 2001). Class I included 10 SgTPS genes, which consisted primarily of diterpene synthases for primary metabolism. Class III included 16 SgTPS genes, which consisted of 11 sesquiterpene synthases (TPS-a) and 5 monoterpene synthases (TPS-g). The size of exons was relatively conserved, with median lengths from 161 to 286 bp, while the intron length varied greatly from 27 to 80,943 bp and most of them being between 76 to 2,000 bp (Supplementary Figure 13).

In addition, three *SgTPS*s from TPS-f clade, *SgTPS59*, *SgTPS63*, and *SgTPS64*, were aligned closely in the same chromosome 1, three from TPS-a, *SgTPS03*, *SgTPS61*, and *SgTPS11*, were aligned closely in chromosome 2, while five *SgTPS*s were aligned closely in chromosome 5 (Figure 4B). Furthermore, according to their expression levels in five tissues, these *SgTPS* genes were divided into five clusters (Figure 4C). Five genes including *SgTPS07*, *SgTPS13*, *SgTPS60*, *SgTPS10*, and *SgTPS14* were expressed at high levels in leaves from the young tree, and four genes, *SgTPS06, SgTPS59, SgTPS56*, and *SgTPS58* were specifically expressed in roots, while three genes *SgTPS62*, *SgTPS03*, and *SgTPS05* were highly expressed in the stem of adult tree and high oleoresin-producing trees (H12). Interestingly, the expression profile of *SgTPS61* was positively correlated with the accumulation rate of terpene content (correlation coefficient = 0.99, *p* < 0.05) (Supplementary Figure 11), whereas *SgTPS46* was negatively correlated with the accumulation rate of terpene content (correlation coefficient = −0.95, *p* < 0.05). These results indicated that SgTPS might have evolved to play diverse and specific roles in the development and defense response of *S. glabra*.

To reveal the potential regulators involved in terpene biosynthesis of *S. glabra*, a weighted correlation network analysis of gene expression in five tissues was performed. We identified 14 modules co-expressed with the accumulation of α-copaene and β-caryophyllene (Supplementary Figure 15), among which yellow and red modules showed significantly positive and negative correlationship with sesquiterpene accumulation, respectively. In the yellow module, four genes encoding rate-limiting enzymes HMGR2, HMGR10, HMGR7, and terpene synthase TPS61, were identified to play essential positive roles in terpene biosynthesis. These genes were co-expressed with transcription factors MYB, HB-HD-ZIP, and AUX (Supplementary Figure 16A). In the red module, the first catalyzing enzyme *ACAT2* in the MVA biosynthesis pathway was co-expressed with large number of transcription factors including bZIP, WRKY, NAC, etc. (Supplementary Figure 16B), and supposed to play key negative roles in terpene accumulation in *S. glabra.*

### Genes Involved in Stress Response of *S. glabra*

**FIGURE 5:**
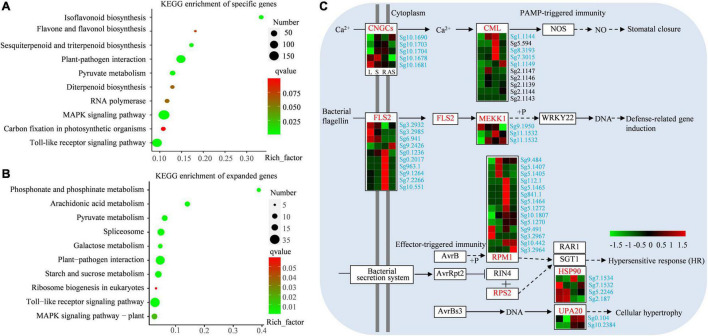
KEGG enrichment and expression analysis of specific and expanded genes in *S. glabra* genome. **(A)** KEGG enrichment analysis of *S. glabra-*specific genes. **(B)** KEGG enrichment analysis of *S. glabra-*expanded genes. **(C)** Expression patterns of specific and expanded genes involved in the plant–pathogen interaction pathway in *S. glabra*. The specific and expanded genes were marked in blue and black, respectively.

Terpenes were reported to be involved in various responses to biotic and abiotic stress. To elucidate the specific functions of terpenes in *S. glabra*, a detailed analysis of *S. glabra* specific and expanded genes were performed. In total, 1,360 genes of *S. glabra*-unique families were annotated using KEGG database, of which 840 were mapped to KEGG pathways. The KEGG pathway assignments were enriched in plant–pathogen interaction (99 genes), MAPK signaling pathway (155 genes), sesquiterpenoid and triterpenoid biosynthesis (14 genes), and diterpenoid biosynthesis (12 genes) (Figure 5A and Supplementary File 3). KEGG enrichment analysis of the 370 expanded genes showed that they were involved in plant–pathogen interaction (31 genes), calcium signaling pathway (12 genes), carbon metabolism (12 genes), and plant MAPK signaling pathway (12 genes) (Figure 5B and Supplementary File 4). These results indicated that the unique and expanded genes might be closely associated with defense response and with high terpene content in *S. glabra*.

Among the unique and expanded genes in the *S. glabra* genome, genes encoding Leaf rust 10 disease-resistance locus receptor-like protein kinase-like (LRK10L) and G-type lectin S-receptor-like serine/threonine-protein kinase (G-LecRK) involved in Toll-like receptor signaling pathway (map04620) were significantly enriched. The *LRK10* gene was firstly cloned from wheat and reported to be involved in disease resistance against the leaf rust-causing fungal pathogen *Puccinia recondite* (Feuillet et al., 1997). We revealed that 17 LRK10Ls underwent expansion and 15 LRK10Ls were unique in the *S. glabra* genome (Supplementary File 11). The G-LecRKs belong to pattern-recognition receptors and engage the recognition of bacteria and fungi for defense response (Vaid et al., 2012). In the *S. glabra* genome 12 G-LecRKs has expanded and 25 G-LecRKs were specific. Moreover, three LRK10Ls and seven G-LecRKs were highly expressed in adult stems of *S. glabra* (Supplementary Figure 17), suggesting potential roles of these genes in biotic defense.

Enrichment analysis also revealed that both the unique and expanded genes were significantly enriched in plant–pathogen interaction pathway (map04626) (Figure 5C). The Ca^2+^-induced defense response is mediated via cyclic nucleotide gated channels (CNGCs), and then the Ca^2+^ sensor calmodulins (CML) interact with downstream effectors that modulate NO production in defense responses (Lecourieux et al., 2006). There were five differentially expressed *S. glabra*-specific *CNGC*s and four unique *CML*s and six expanded *CML*s in the *S. glabra* genome. The bacterial flagellin was recognized by receptor-like kinase flagellin-sensitive22 (FLS2) and activate pathogen-associated molecular pattern (PAMP)-triggered immunity through a mitogen-activated protein kinase kinase kinase 1 (MEKK1) (Liu et al., 2013). Ten *FLS2* and three *MEKK1* genes were identified as *S. glabra*-specific genes. Disease resistance (R) protein NBS-LRR family encode proteins containing a nucleotide binding site (NBS) and carboxyl-terminal leucine-rich repeats (LRRs) and specifically recognizes the AvrB and AvrRpt2 effectors, respectively (Mackey et al., 2003). Overall, 245 genes with an NBS domain were identified in the *S. glabra* genome (Supplementary File 12). Among these genes, there were 17 and 47 *NBS-LRR*s identified as expanded and specific genes, respectively. Phylogenetic analysis revealed that *NBS-LRR* family underwent a lineage-specific expansion in *S. glabra* genome (Supplementary Figure 18A). These *NBS-LRRs* exhibited different expression patterns, with the majority of them being highly expressed in roots and mature stems (Supplementary Figure 18B), indicating specialized defense functions in various tissues. The heat shock protein HSP90 is required for full RPS2 resistance. Four *HSP90* genes were unique in *S. glabra* genome. Effector AvrBs3 induces the expression of a BHLH transcription factor UPA20 and causes cellular hypertrophy (Takahashi et al., 2003). Two *UPA20* genes were found to be specific in the *S. glabra* genome.

## Discussion

Rapid development and advancement of new sequencing technologies in recent years led to a sharp increase in the number of plant genomes being sequenced. But most sequenced plant genomes are restricted to a narrow phylogenetic range. Moreover, the focus of genome research in Leguminosae has been primarily limited to crop and herbaceous plants in the Papilionoideae subfamily, such as *C. arietinum* (Varshney et al., 2013), *M. truncatula* (Young et al., 2011), *Lotus japonicas* (Sato et al., 2008), and *A. duranensis* (Bertioli et al., 2016). To our knowledge, few studies were conducted on the plants from Caesalpinioideae and Mimosaceae subfamilies (Griesmann et al., 2018; Chang et al., 2019). Here we reported the whole genome of *S. glabra* tree from the subfamily Caesalpinoideae using a combined strategy with Illumina short read sequencing and PacBio long-read sequencing. Many assisted genome assembly were performed combing Hi-C, 10× Genomics, BioNano and genetic maps (Peng et al., 2019; Chen et al., 2020). In this study, Hi-C technology was applied to anchor the genomic sequences to 12 chromosomes, which was consistent with the number of chromosomes reported in *Sindora* Miq. (Goldblatt, 1981). Besides, according to BUSCO, CEGMA and transcriptome data analysis, the final genome of *S. glabra* is quite complete and accurate, which provided useful resources for further functional analysis.

The final assembled genome size was 1.1 Gb with a contig N50 of 1.3 Mb. The genome size of *S. glabra* was comparable to that of *G. max* (1.1 Gb) (Schmutz et al., 2010), *Lupinus angustifolius* (1.2 Gb) (Yang et al., 2013), and *A. duranensis* (1.3 Gb) (Bertioli et al., 2016), but about two times bigger than other sequenced plant genomes in the Fabaceae family such as *M. truncatula* (454 Mb) (Young and Bharti, 2012), *V. unguiculata* (519 Mb) (Lonardi et al., 2019), *Lotus japonicus* (472 Mb) (Sato et al., 2008), and *Faidherbia albida* (661 Mb) (Chang et al., 2019). The genome size differences among Fabaceae species may be attributable to changes in the amount of retrotransposons. The *S. glabra* genome contained 11.6% of *Gypsy* and 12.9% of *Copia*, while *L. japonicas* contained 8.81% of *Gypsy*, and 7.16% of *Copia* (Sato et al., 2008). Differential amplification recently, or differential retention of ancient insertions might have resulted in transposon abundance, and eventually leading to whole genome size expansion in *S. glabra.* This is also observed between *Vigna* species (Lonardi et al., 2019).

Whole genome duplication analysis revealed that three Fabaceae plants *S. glabra, G. max*, and *M. truncatula* all undergone early-legume genome duplication, while *C. micranthum* from Lauraceae family only shares an ancient duplication event (Figure 2C). The sequenced genomes of agriculturally important legume plants mostly belong to Papilionoideae subfamily and comparison among these Papilionoideae genomes reveal a whole-genome duplication event approximately 58 mya (Young and Bharti, 2012). The existence of whole-genome duplication in the *S. glabra* from Caesalpinioideae subfamily further supported that legume plants share ancient whole genome duplication, which could contribute to the origin of novel key traits and drove species diversification. Therefore, it was postulated that *S. glabra* may have undergone recent whole-genome duplication after the divergence from Papilionoideae family 73.3 mya (Figure 2B). Moreover, *S. glabra* genome harbors 31,944 genes, which is much more than the number of protein-coding genes in *C. micranthum* (27,899 genes) (Chaw et al., 2019). The recent genome duplication may lead to more genes in *S. glabra* than in *C. micranthum* (Schranz et al., 2012). Furthermore, the largest chromosome 1 of *S. glabra* exhibited no collinear intervals with other leguminous plants (Figure 2D), which might indicate lineage-specific evolution of genes in *S. glabra* genome.

Gene family comparison among 16 different species identified 1,035 gene families that are specific to *S. glabra.* These genes were mainly involved in signal transduction, phloem development and terpene synthase activity (Supplementary File 4). Terpene synthases TPSs are the key enzymes responsible the biosynthesis of terpenes. They are widely distributed in plant species with the family members ranging from 2 to 79 full-length *TPS*s, except algae (Jiang et al., 2019). We performed a genome-wide analysis of *TPS* genes in *S. glabra* genome and revealed 64 putative *SgTPS*s, which number is much more than other Fabaceae plants such as *M. truncatula* (40 *TPS*s) and *G. max* (30 *TPS*s) (Jiang et al., 2019). According to phylogenetic analysis, 34 and 31% of *SgTPS*s were classified in TPS-a1 and TPS-e/f subfamily, respectively. The TPS-a1 subfamily encodes only sesquiterpenes that are found in dicot plants, while TPS-e/f subfamily encodes copalyl diphosphate synthases and kaurene synthases, responsible for gibberellic acid biosynthesis. In addition, these SgTPSs tend to cluster together in each subfamily, suggesting lineage-specific expansion. Therefore, the large number of *TPS* genes in *S. glabra* genome might be attributable to large scale of family expansion during species divergence. Furthermore, there were 11 *SgTPS*s (34%) identified as tandemly duplicated genes (Figure 4B), indicating that tandem duplication plays an important role in the subfamily expansion of SgTPSs.

*Sindora glabra* tree accumulates abundant amount of oleoresin, among which α-copaene and β-caryophyllene accounted for about 55% of all sesquiterpenes (Yu et al., 2018). Identification and characterization of *S. glabra* terpene biosynthesis genes is essential for improving oleoresin production. There were 59 terpene backbone biosynthesis genes identified in the *S. glabra* genome, among which *HMGR* gene family in the MVA pathway contained the largest number of genes (10 *HMGR*s), followed by *DXS*, *FPS*, and *GGPS* with seven genes (Figure 3). The number of *HMGR*s in the *S. glabra* genome was much more than that in other oil-producing plants including *E. ulmoides* (four *HMGR*s) (Wuyun et al., 2018), and *Litsea cubeba* (four *HMGR*s) (Chen et al., 2020). In *E. ulmoides*, the rubber biosynthesis was mainly attributable to the expansion of *FPS* and small rubber particle protein (*SRPP*) genes in the MVA pathway. In *L. cubeba*, the expansion of *DXS* and mono-*TPS* genes contributed to the biosynthesis of large amounts of monoterpenes in the flowers. Furthermore, the expression levels of three *HMGR*s and one *TPS* were positively correlated with the accumulation patterns of sesquiterpene in the *S. glabra* stems. These suggested that the expansion of *HMGR*s and *TPS*s in the *S. glabra* genome is important for the copious production of sesquiterpenes in the tree trunk. Therefore, it was proposed that the amount and types of oils produced in various species may be directly related to the number and expression levels of genes involved in oil biosynthesis.

The regulatory factors involved in terpene biosynthesis have been identified in various species. However, due to limited genomic information in some oil-producing plants, these identified transcription factors are mostly homologous to model plants and largely distributed in the WRKY, MYC, ERF, and MYB families (Xu et al., 2004; Yu et al., 2012; Shen et al., 2016; Jian et al., 2019). Here we performed a comprehensive gene co-expression analysis using data from five tissues of *S. glabra*. Two significant modules were identified to be related to the sesquiterpene accumulation. In the yellow module, four genes including *TPS61* and three *HMGR*s, were found as core genes for promoting terpene biosynthesis, while in the red module three genes including one *ACAT2* and two other *HMGR*s were identified as core genes for abating terpene production. The co-expressed transcription factors included MYB, NAC, WRKY, and ERF, which were previously reported homologous genes. Besides, most of co-expressed transcription factors have not been reported to be involved in the regulation of terpene biosynthesis in plants such as HB-HD-ZIP, CAMTA, bHLH, and so on. HD-ZIP members have been shown to repress anthocyanin biosynthesis in both Arabidopsis and apple (LaFountain and Yuan, 2021). CAMTA3 was found to mediate biotic defense responses in Arabidopsis (Galon et al., 2008). Jasmonate could mediate anthocyanin accumulation through bHLH/MYB complex in Arabidopsis (Qi et al., 2011). In *S. glabra*, jasmonate was discovered to promote terpene biosynthesis in stems (Yu et al., 2020a). It was proposed that jasmonate might regulate terpene biosynthesis through bHLH/MYB interactions. The comprehensive gene co-expression networks provide new insights into terpene biosynthesis by revealing multiple regulatory pathways. However, whether these transcription factors act directly or indirectly and how their hierarchical network was formed to regulate terpene biosynthesis need further substantial efforts in future.

Oleoresin terpene in conifers plays an important defense function against herbivores and pathogens (Celedon and Bohlmann, 2019). However, the oleoresins in conifers are mainly composed of monoterpenes and diterpenes, while the oleoresins in broadleaf tree *S. glabra* are largely composed of sesquiterpenes. In the broadleaf trees, research on terpene function in the genus *Eucalyptus* is relatively well developed. Terpenes released from *Eucalyptus* leaves could protect themselves against the most damaging pest, *Leptocybe invasa* (Oates et al., 2015). Terpenes constituents of anther in *Eucalyptus polybractea* have multiple roles including attracting pollinators, deterrence of palynivores and adhesion of pollen to pollinators (Goodger et al., 2021). Nevertheless, although there were a small amount of sesquiterpenes detected in *Eucalyptus*, monoterpenes still constituted the majority of terpenes. It is difficult to determine the function of specific compounds or if the synergistic effect of different types of terpenes is needed for their defenses. In *S. glabra* trunk, above 80% of oleoresin was composed of sesquiterpenes that share the same molecular weight. Studies on the function of sesquiterpene in *S. glabra* trunk would be important for understanding the defense mechanisms in long-lives woody trees. Here, we revealed that the unique and expanded genes in the *S. glabra* genome were mostly enriched in the plant–pathogen interaction and terpene biosynthesis pathways (Figure 5). Specifically, 245 disease resistance genes with an NBS domain were identified in the *S. glabra* genome, which number was much more than that in *Vernicia fordii* (88) (Zhang et al., 2019), but similar to that in *Ricinus communis* (232) (Chan et al., 2010), and *J. curcas* (275) (Wu et al., 2015), and markedly lower than that in *H. brasiliensis* (483) (Lau et al., 2016). The NBS-LRR family gene showed lineage-specific expansion to give 47 members. In addition, the expression of these disease resistance genes showed diverse expression patterns and some of them were highly expressed in the high oil-yielding adult stem tissues. Together, these suggest that *S. glabra* may have evolved complex oleoresin terpene defense mechanism through lineage-specific expansion of terpene biosynthesis genes and disease resistance genes.

In summary, this study presents the whole-genome sequences with comprehensive annotations, including complete pictures of terpene biosynthesis and defense pathways in *S. glabra*. These data will offer valuable resources for rational exploitation and usage of tree oleoresin.

## Data Availability Statement

The datasets presented in this study can be found in online repositories. The names of the repository/repositories and accession number(s) can be found below: https://www.ncbi.nlm.nih.gov/bioproject/PRJNA747876.

## Author Contributions

NY conceived and performed the project and wrote the manuscript. HS helped manuscript revision. JY provided the plant materials for sequencing. RL discussed the results. All authors contributed to the article and approved the submitted version.

## Conflict of Interest

The authors declare that the research was conducted in the absence of any commercial or financial relationships that could be construed as a potential conflict of interest.

## Publisher’s Note

All claims expressed in this article are solely those of the authors and do not necessarily represent those of their affiliated organizations, or those of the publisher, the editors and the reviewers. Any product that may be evaluated in this article, or claim that may be made by its manufacturer, is not guaranteed or endorsed by the publisher.

## Footnotes

1
http://www.timetree.org/
