# Supplementary material for: The Diesel Tree Sindora glabra Genome Provides Insights Into the Evolution of Oleoresin Biosynthesis
Source: Front Plant Sci. 2022 Jan 4;12:794830. doi: 10.3389/fpls.2021.794830 (PMC8764381; doi:10.3389/fpls.2021.794830)
Supplement: Supplementary file 12 [file Presentation_1.PPTX]

## Slide 1
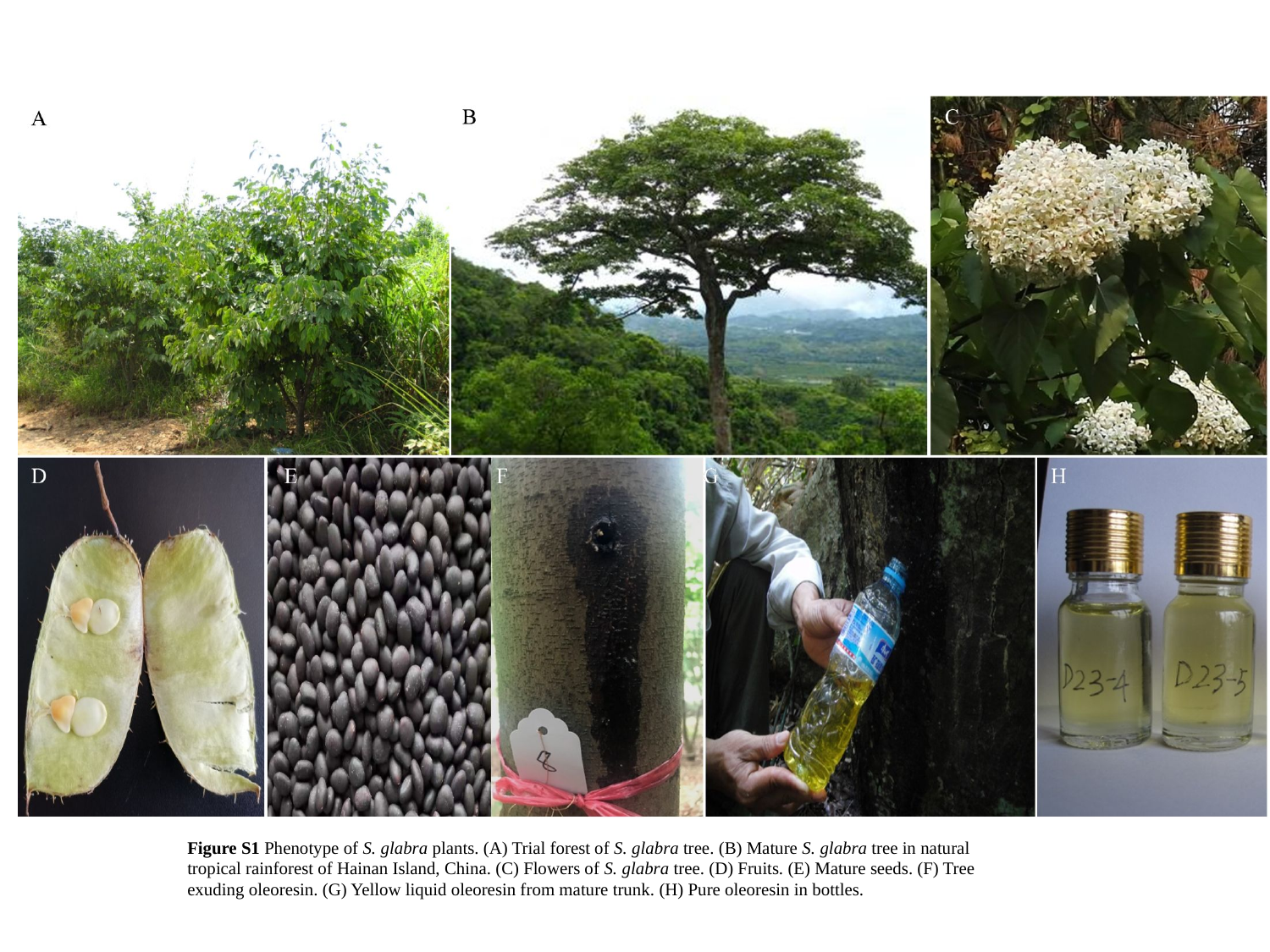

Figure S1 Phenotype of S. glabra plants. (A) Trial forest of S. glabra tree. (B) Mature S. glabra tree in natural tropical rainforest of Hainan Island, China. (C) Flowers of S. glabra tree. (D) Fruits. (E) Mature seeds. (F) Tree exuding oleoresin. (G) Yellow liquid oleoresin from mature trunk. (H) Pure oleoresin in bottles.

## Slide 2
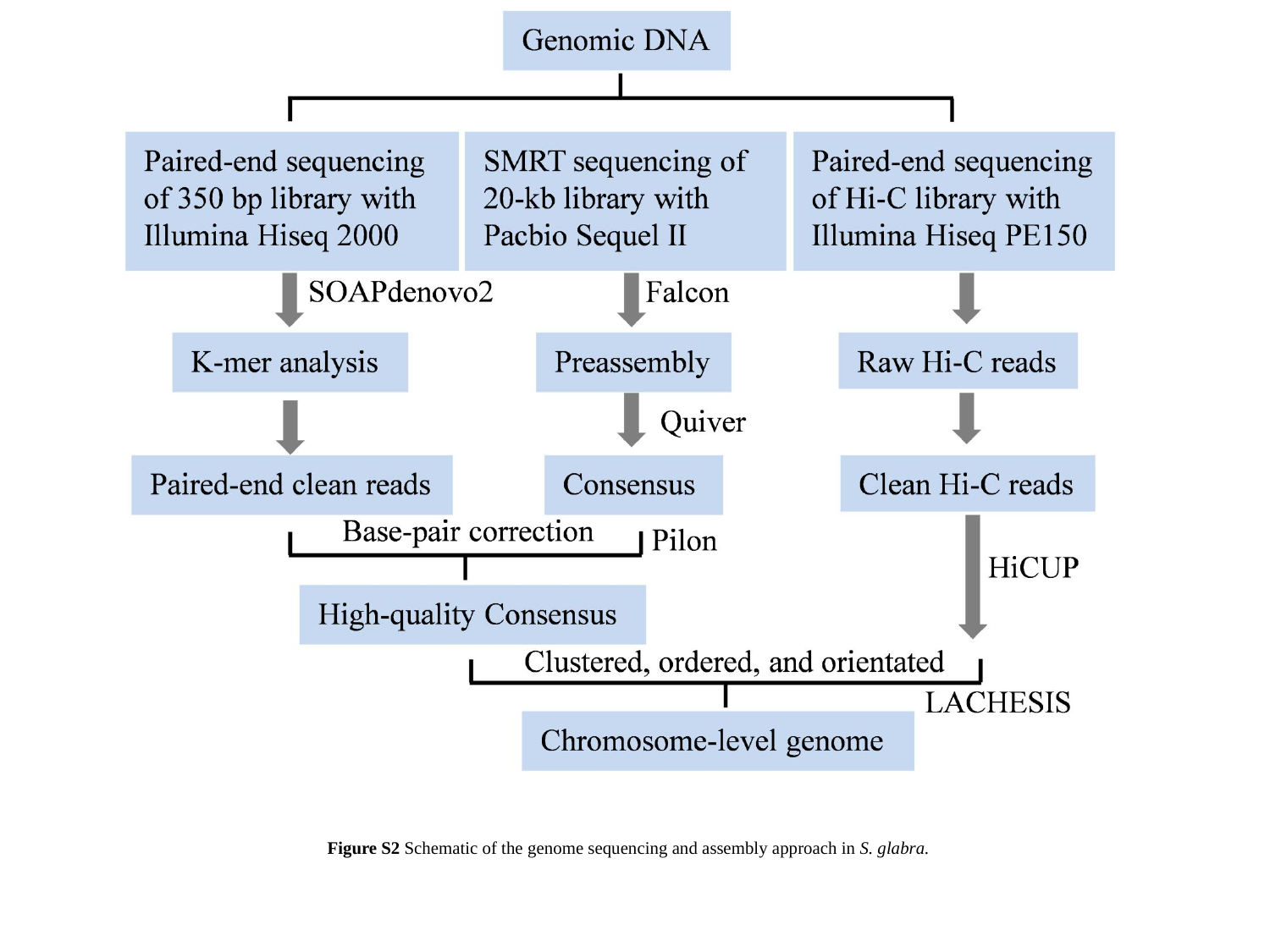

Figure S2 Schematic of the genome sequencing and assembly approach in S. glabra.

## Slide 3
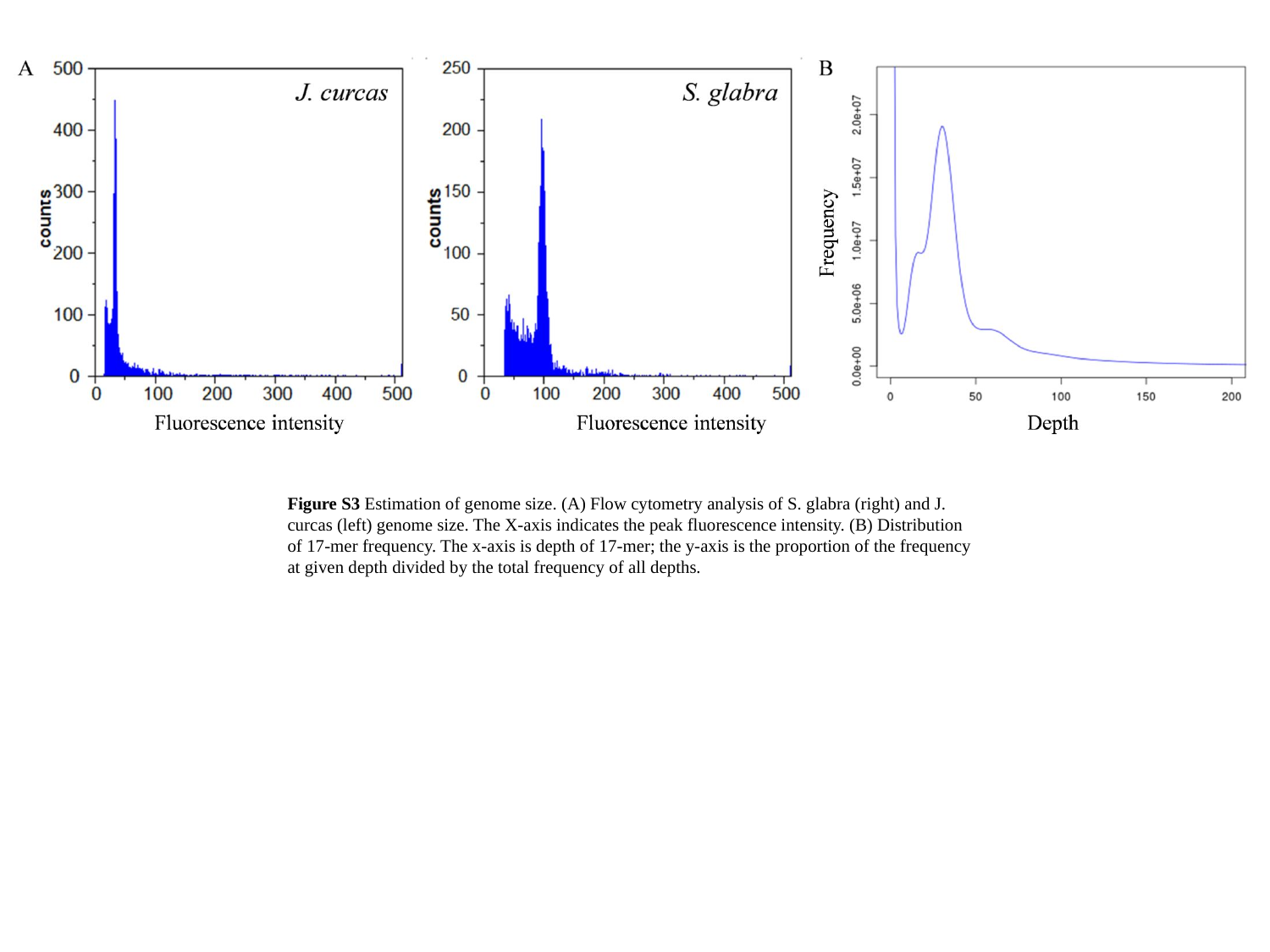

Figure S3 Estimation of genome size. (A) Flow cytometry analysis of S. glabra (right) and J. curcas (left) genome size. The X-axis indicates the peak fluorescence intensity. (B) Distribution of 17-mer frequency. The x-axis is depth of 17-mer; the y-axis is the proportion of the frequency at given depth divided by the total frequency of all depths.

## Slide 4
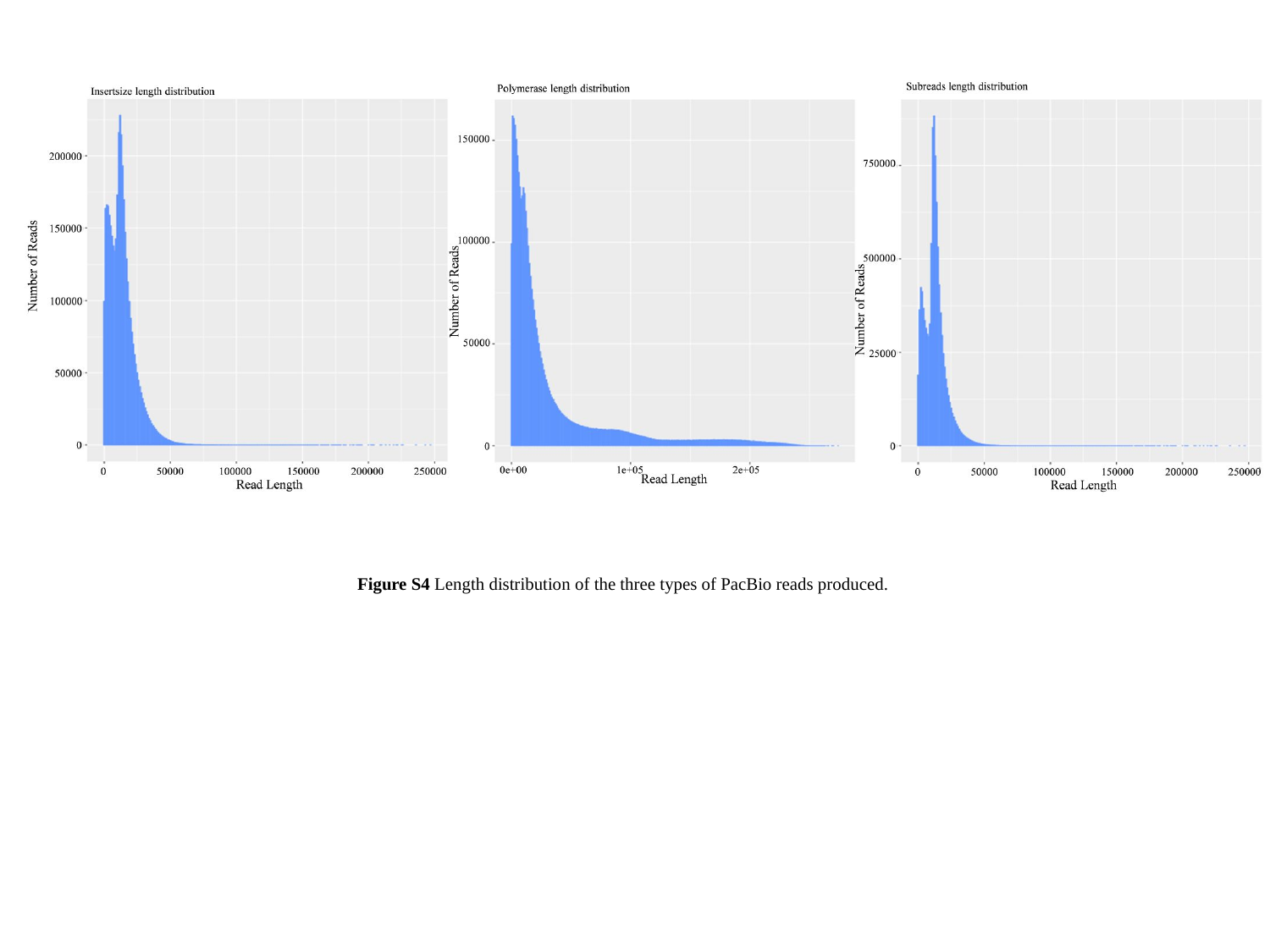

Figure S4 Length distribution of the three types of PacBio reads produced.

## Slide 5
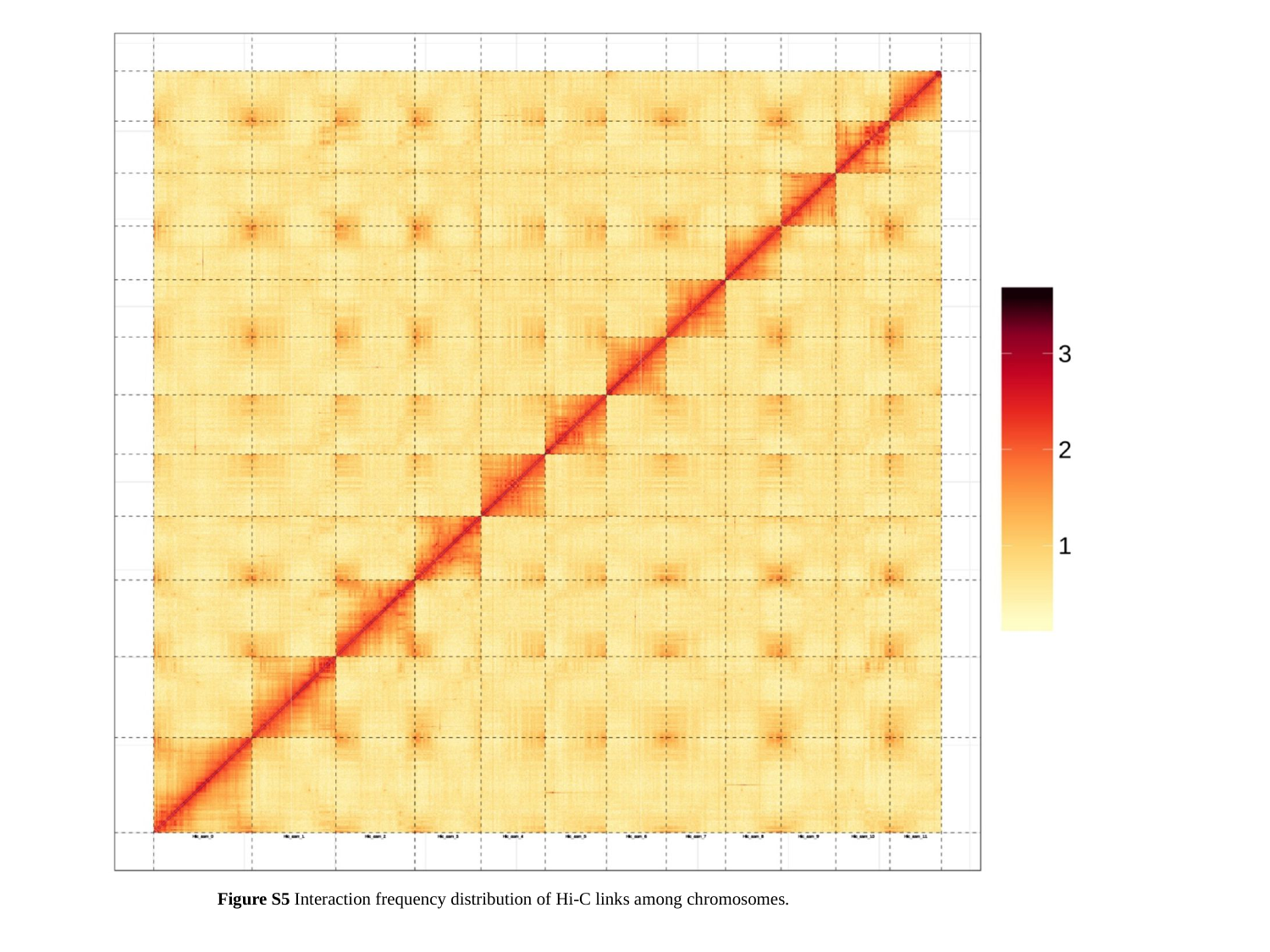

Figure S5 Interaction frequency distribution of Hi-C links among chromosomes.

## Slide 6
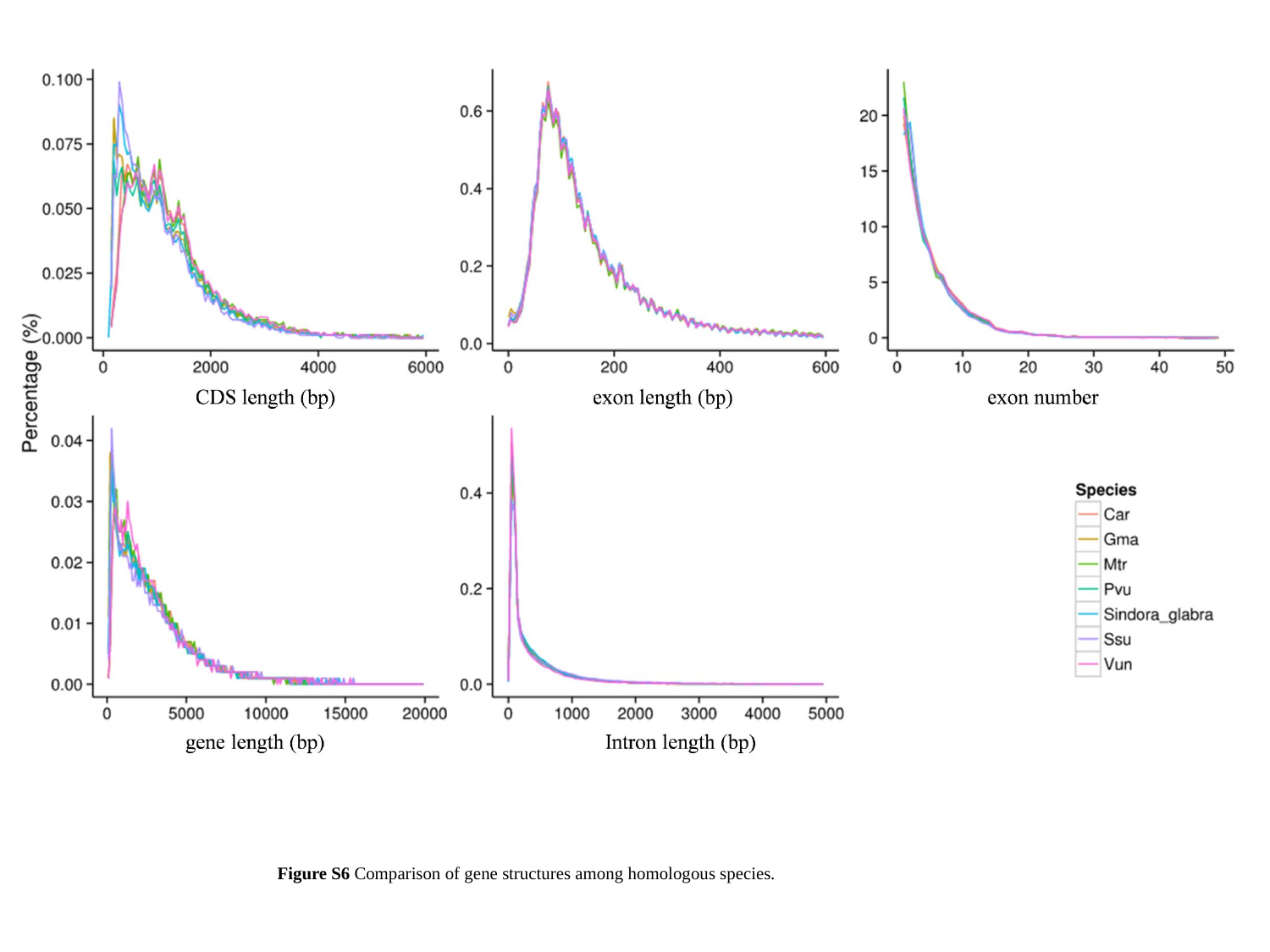

Figure S6 Comparison of gene structures among homologous species.

## Slide 7
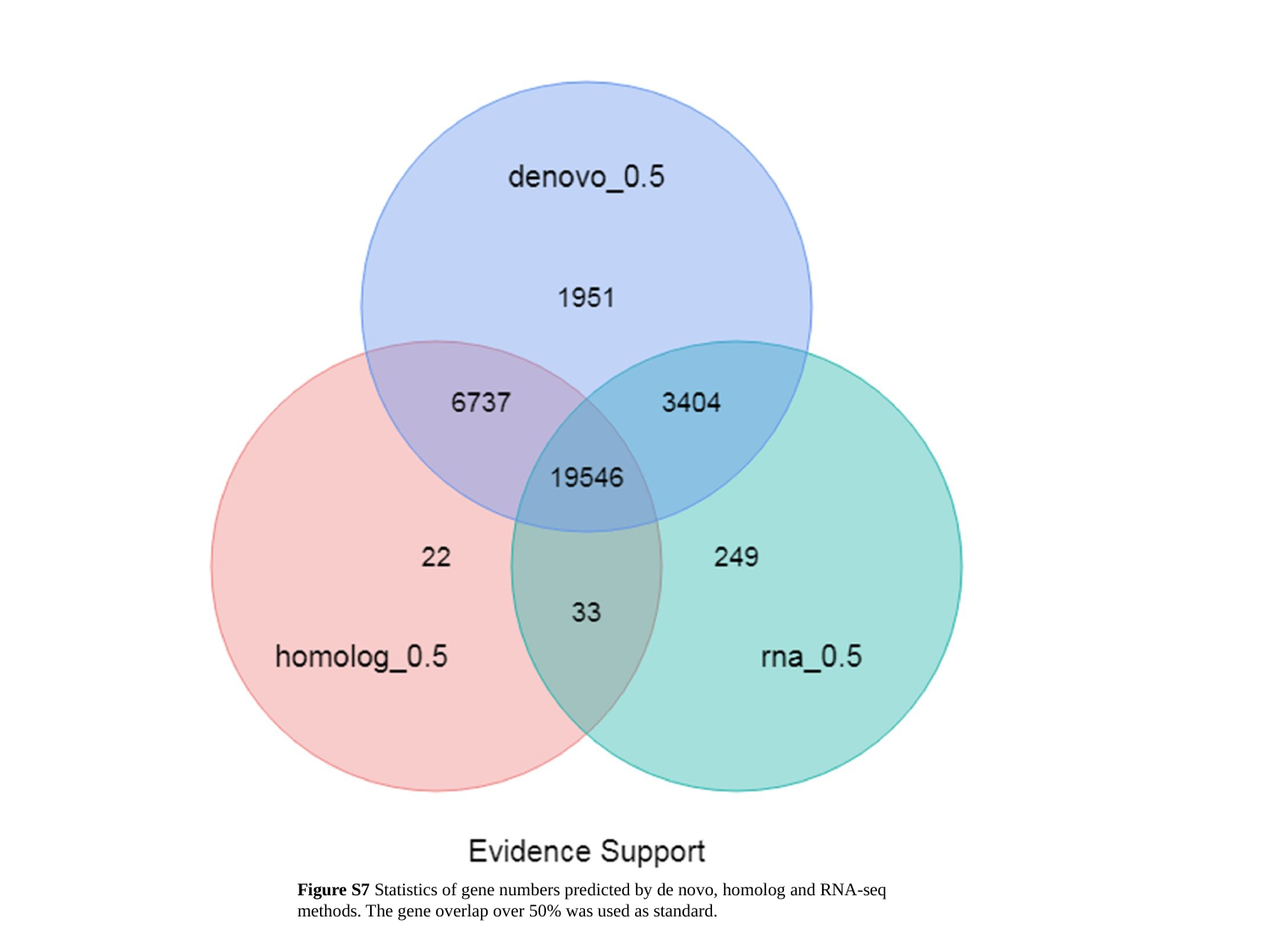

Figure S7 Statistics of gene numbers predicted by de novo, homolog and RNA-seq methods. The gene overlap over 50% was used as standard.

## Slide 8
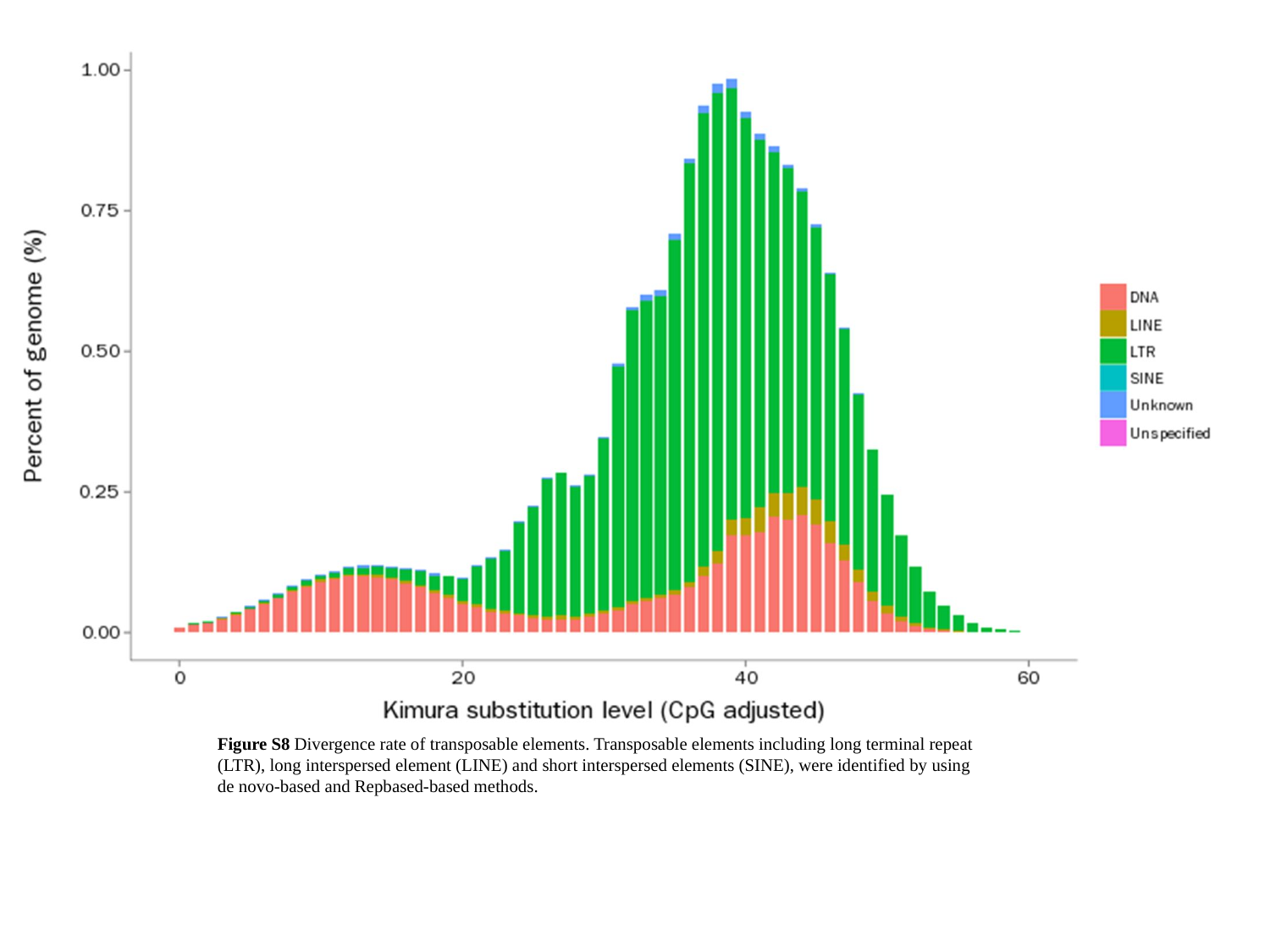

Figure S8 Divergence rate of transposable elements. Transposable elements including long terminal repeat (LTR), long interspersed element (LINE) and short interspersed elements (SINE), were identified by using de novo-based and Repbased-based methods.

## Slide 9
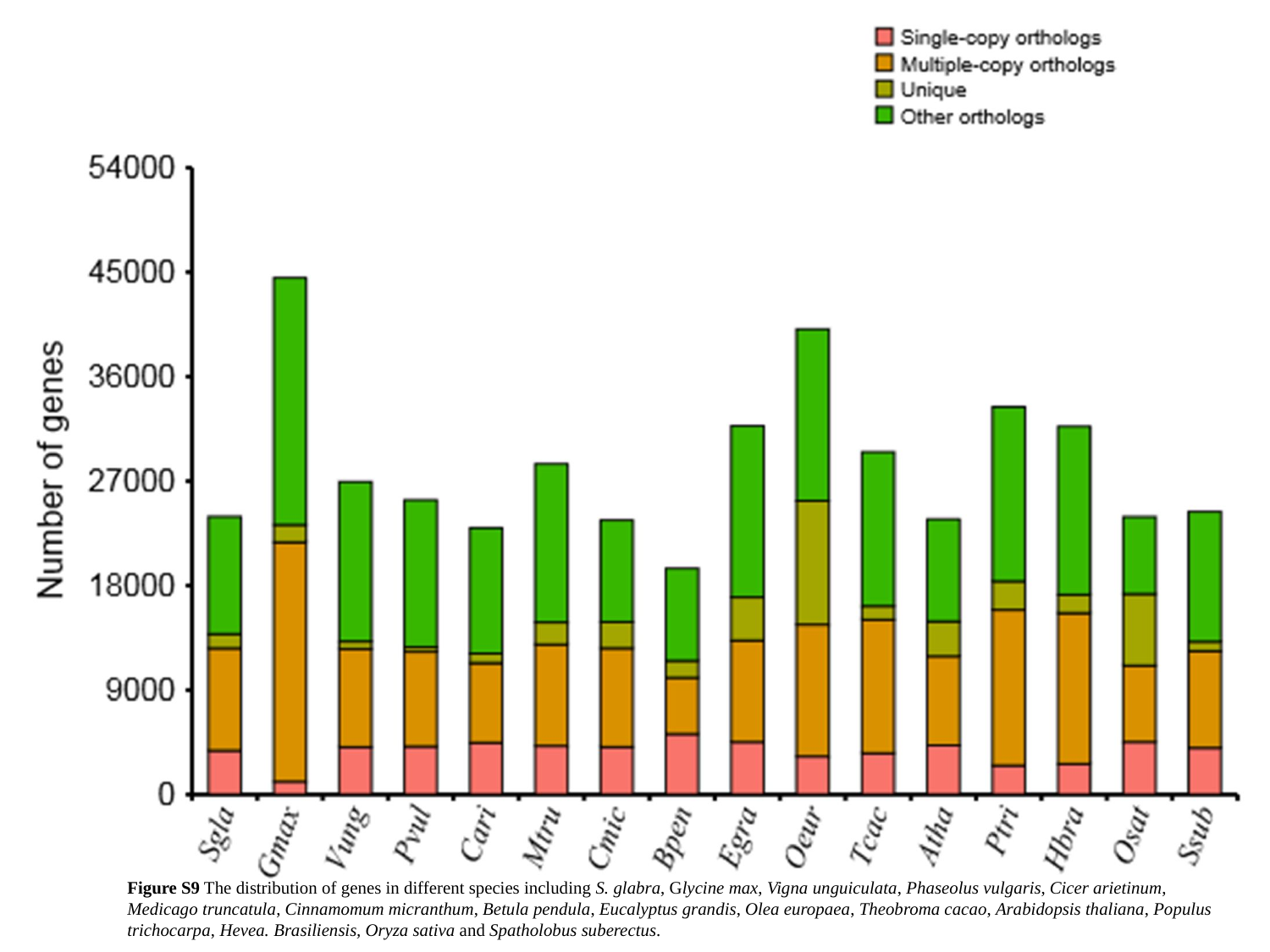

Figure S9 The distribution of genes in different species including S. glabra, Glycine max, Vigna unguiculata, Phaseolus vulgaris, Cicer arietinum, Medicago truncatula, Cinnamomum micranthum, Betula pendula, Eucalyptus grandis, Olea europaea, Theobroma cacao, Arabidopsis thaliana, Populus trichocarpa, Hevea. Brasiliensis, Oryza sativa and Spatholobus suberectus.

## Slide 10
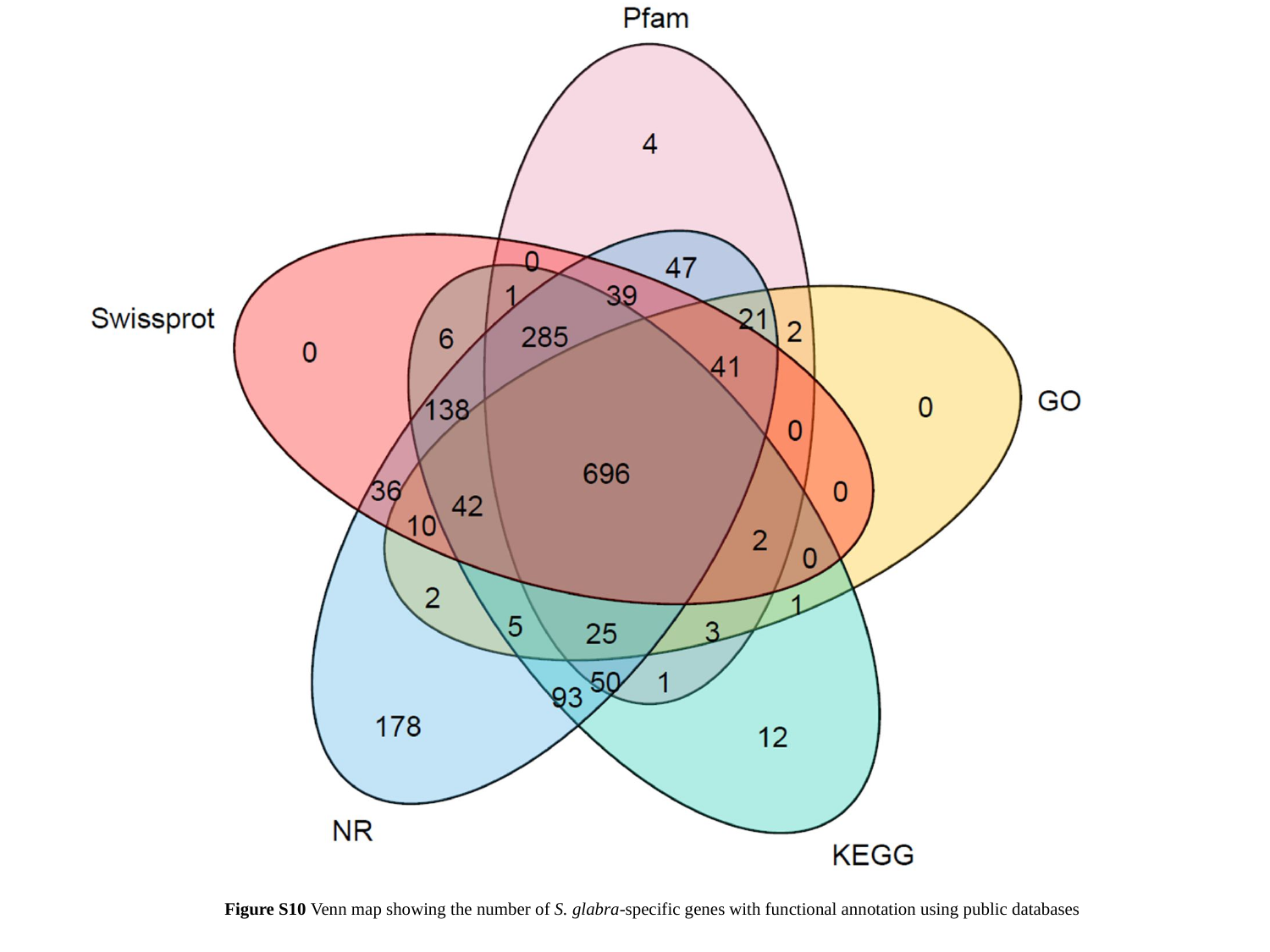

Figure S10 Venn map showing the number of S. glabra-specific genes with functional annotation using public databases

## Slide 11
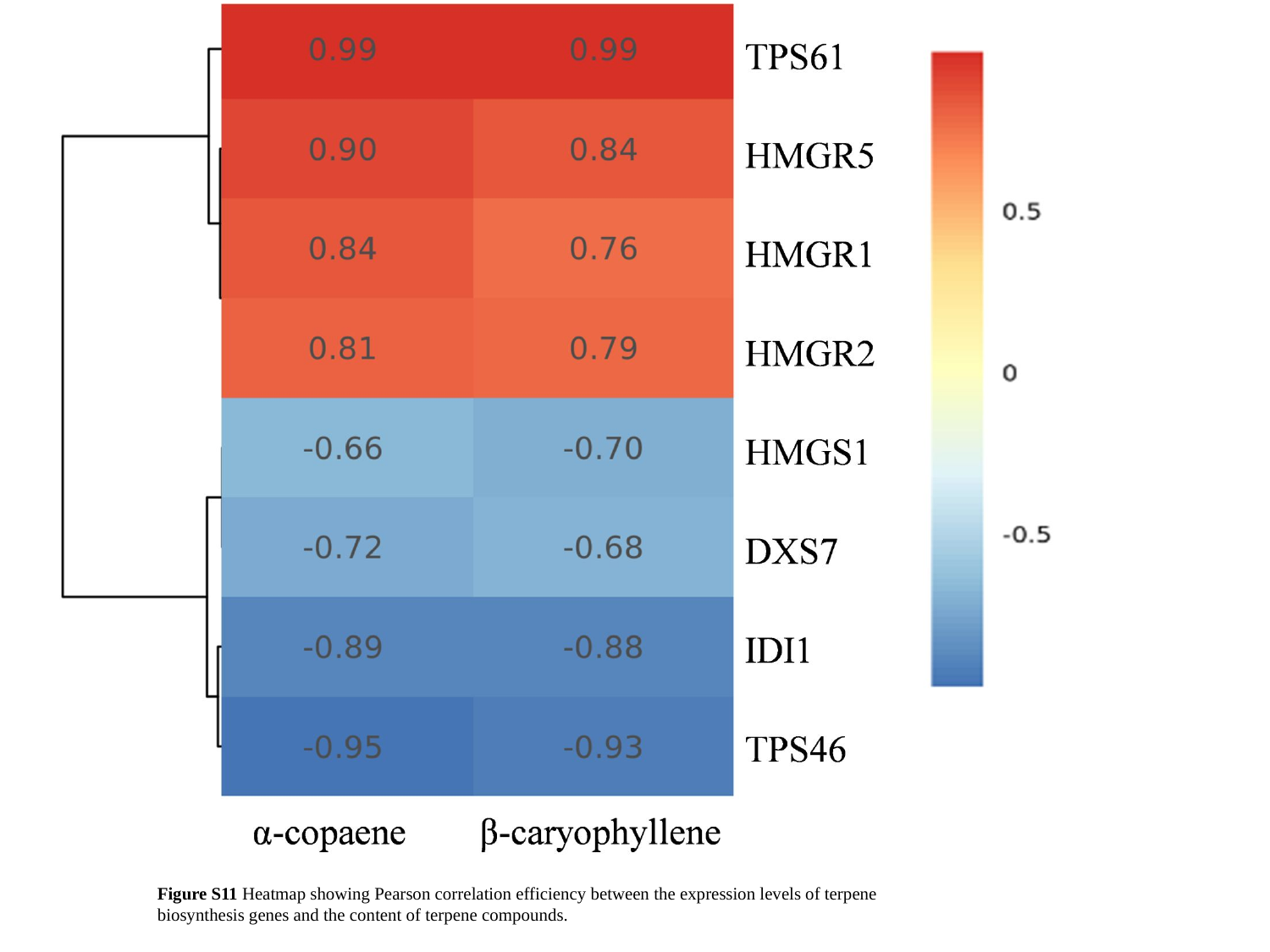

Figure S11 Heatmap showing Pearson correlation efficiency between the expression levels of terpene biosynthesis genes and the content of terpene compounds.

## Slide 12
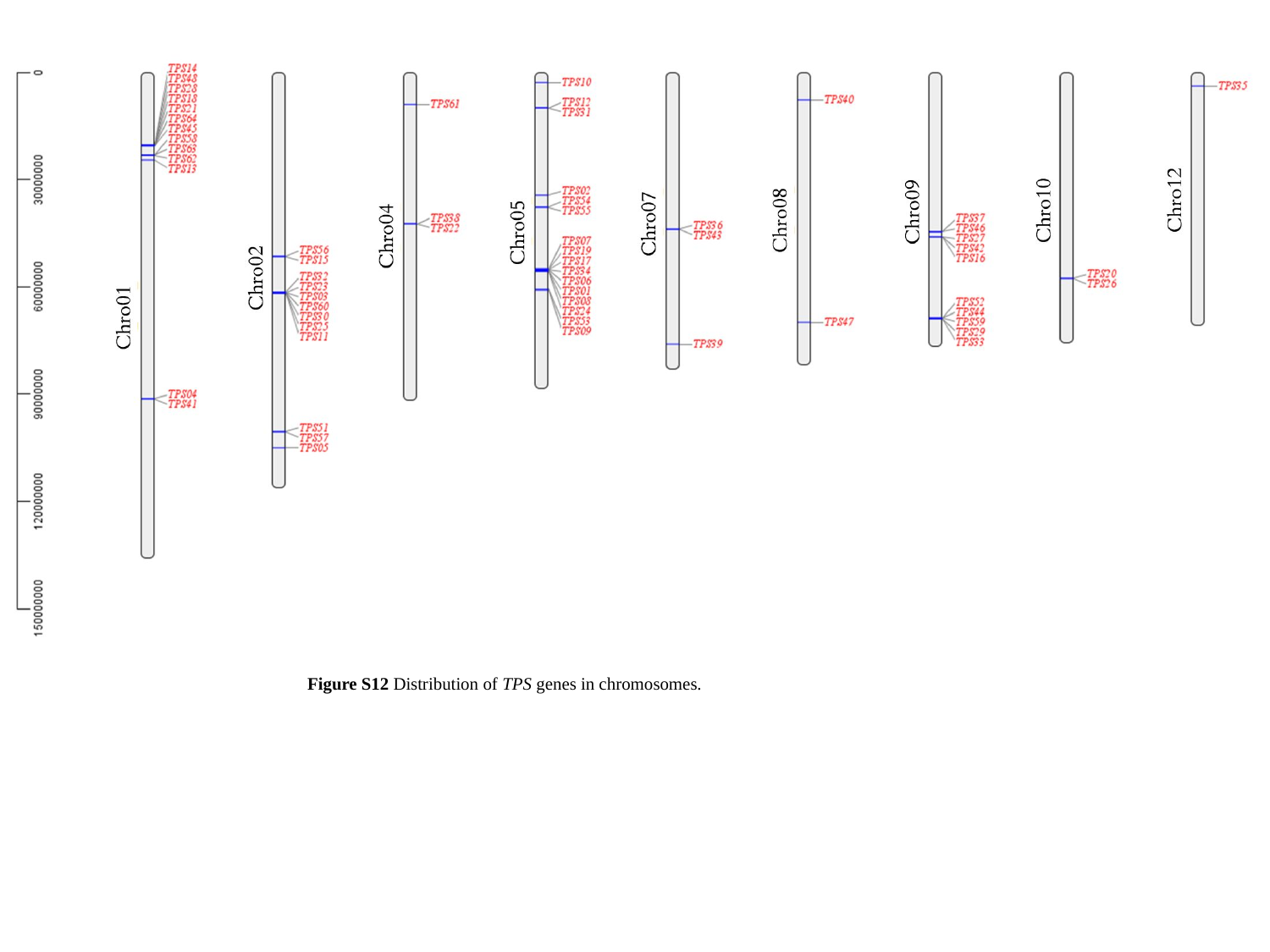

Figure S12 Distribution of TPS genes in chromosomes.

## Slide 13
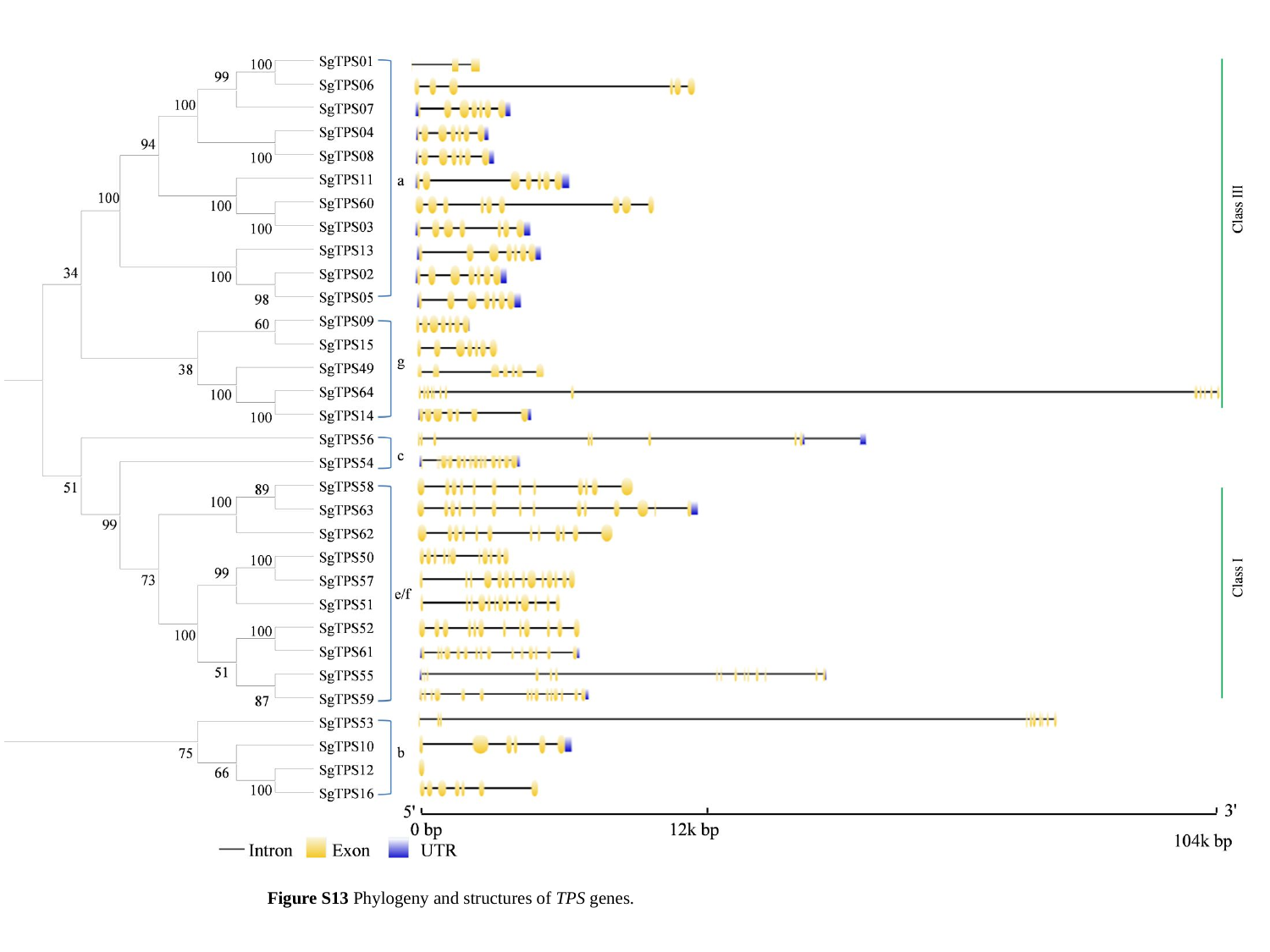

Figure S13 Phylogeny and structures of TPS genes.

## Slide 14
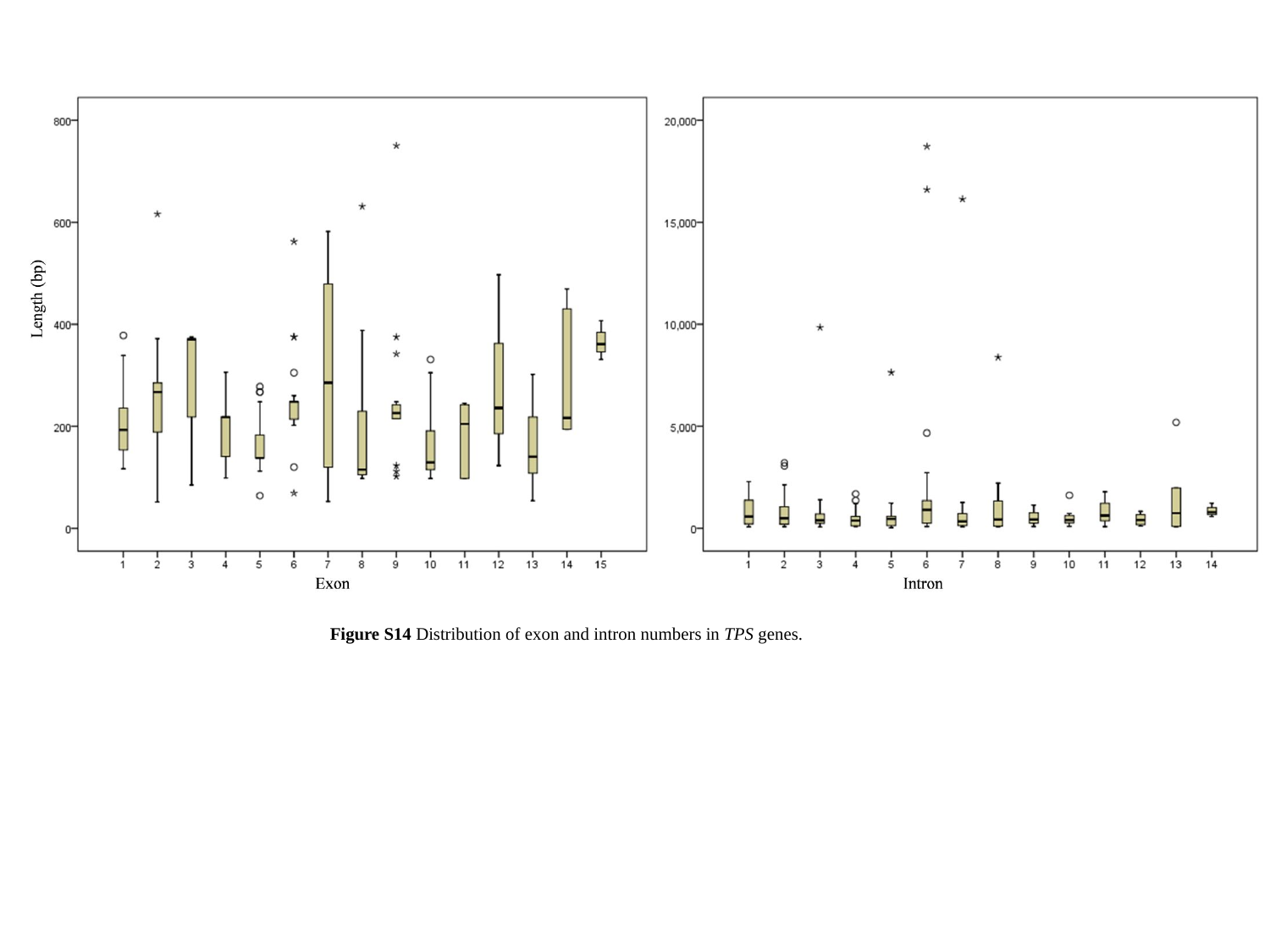

Figure S14 Distribution of exon and intron numbers in TPS genes.

## Slide 15
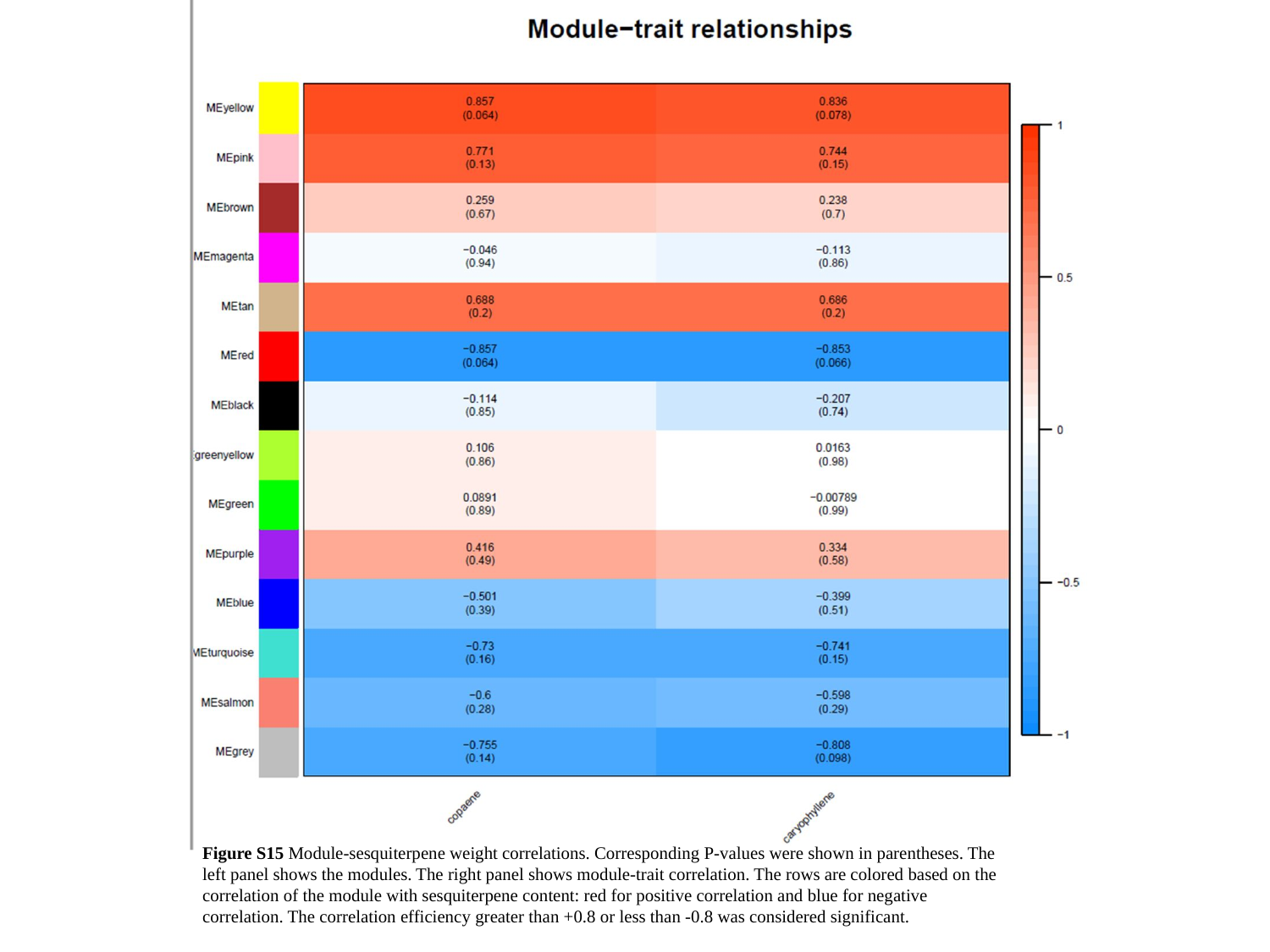

Figure S15 Module-sesquiterpene weight correlations. Corresponding P-values were shown in parentheses. The left panel shows the modules. The right panel shows module-trait correlation. The rows are colored based on the correlation of the module with sesquiterpene content: red for positive correlation and blue for negative correlation. The correlation efficiency greater than +0.8 or less than -0.8 was considered significant.

## Slide 16
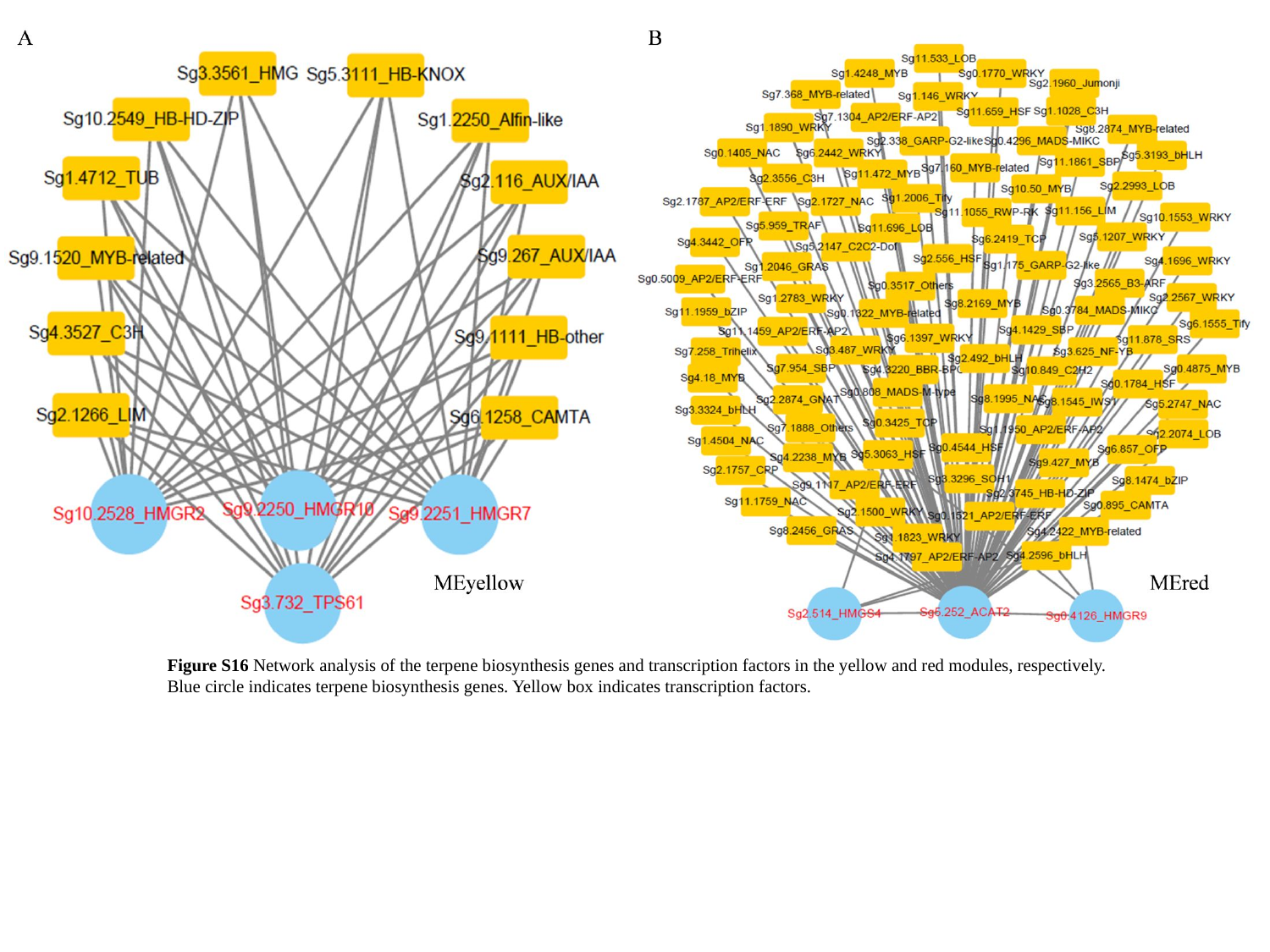

Figure S16 Network analysis of the terpene biosynthesis genes and transcription factors in the yellow and red modules, respectively. Blue circle indicates terpene biosynthesis genes. Yellow box indicates transcription factors.

## Slide 17
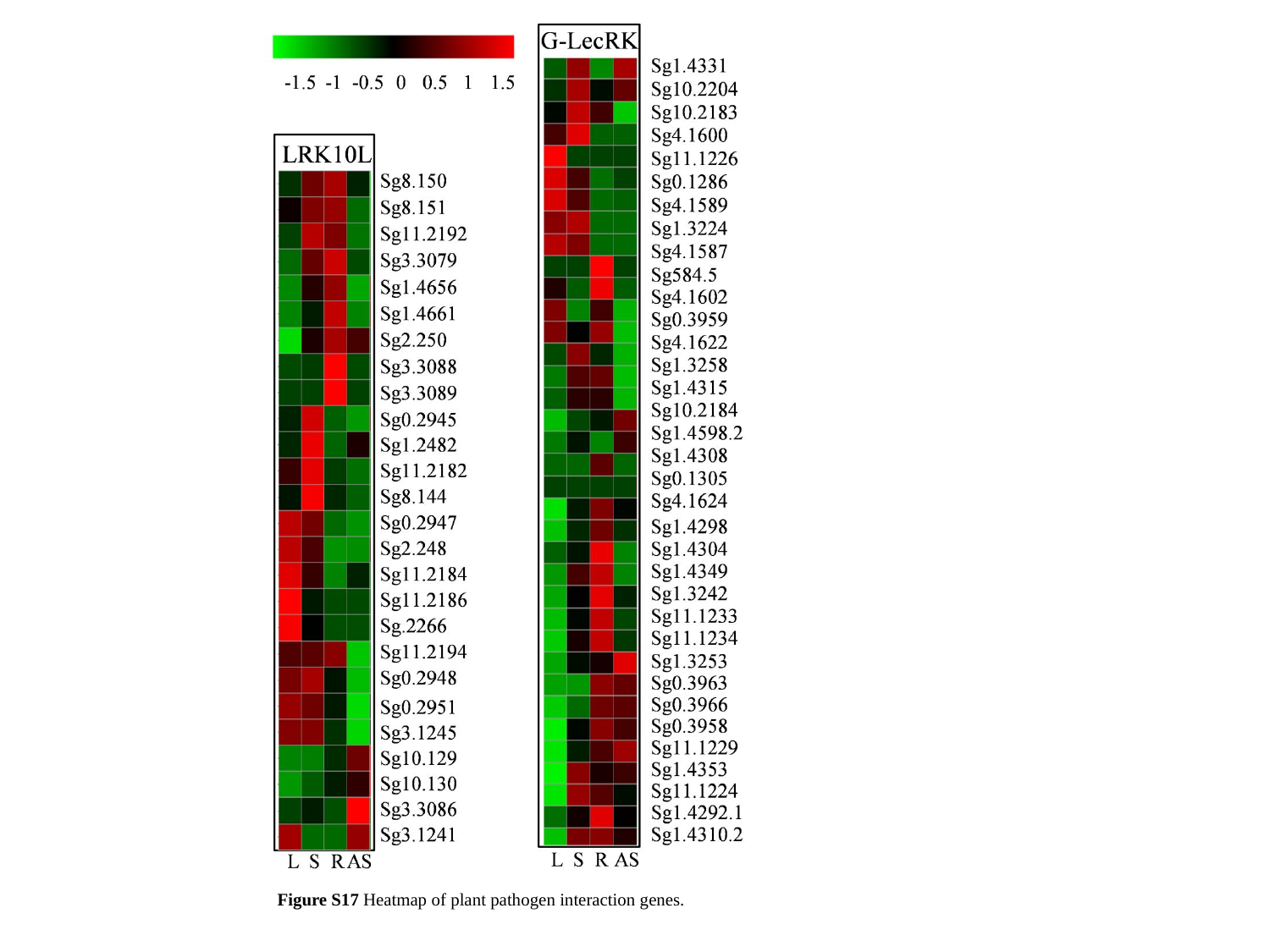

Figure S17 Heatmap of plant pathogen interaction genes.

## Slide 18
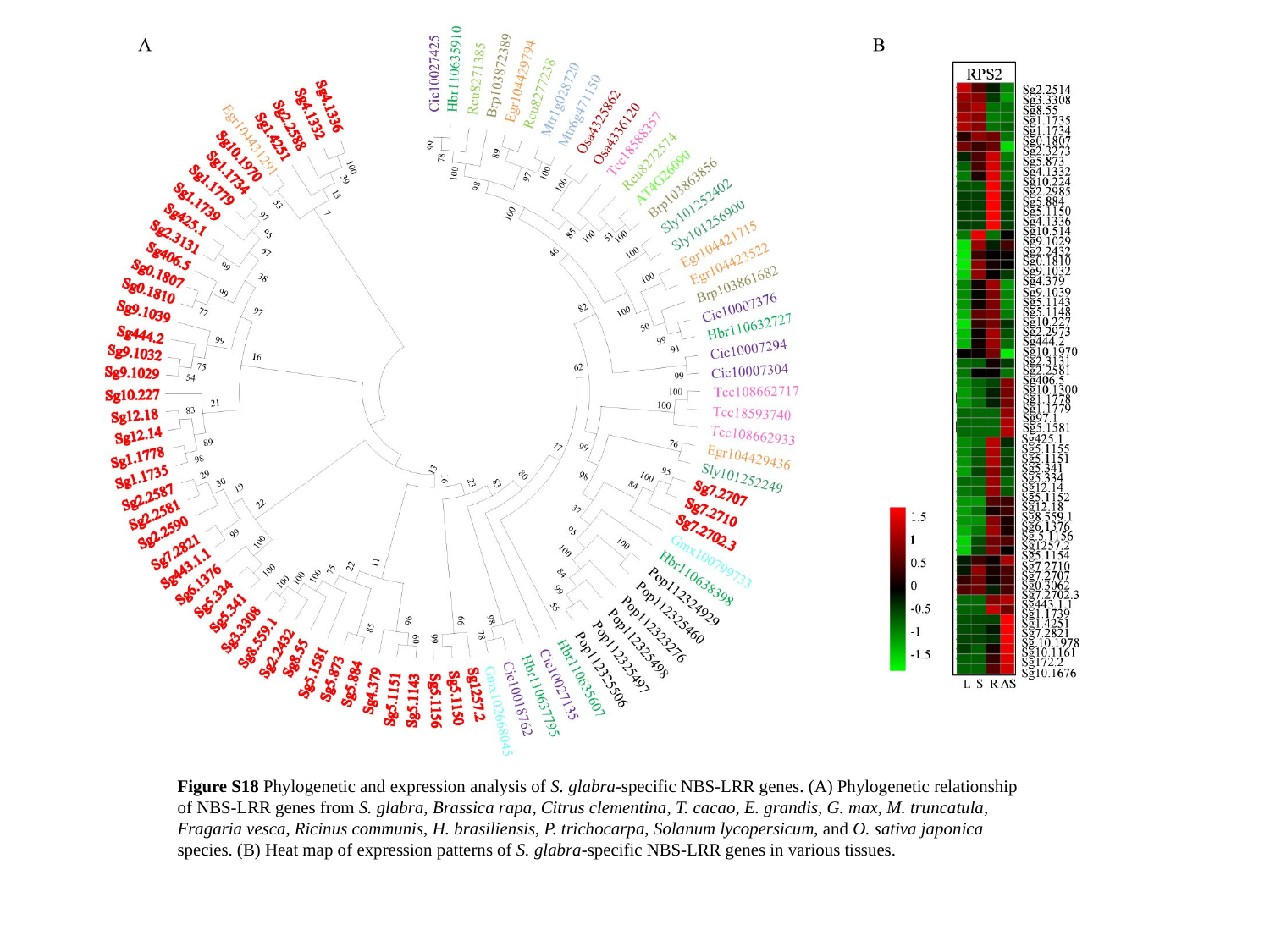

Figure S18 Phylogenetic and expression analysis of S. glabra-specific NBS-LRR genes. (A) Phylogenetic relationship of NBS-LRR genes from S. glabra, Brassica rapa, Citrus clementina, T. cacao, E. grandis, G. max, M. truncatula, Fragaria vesca, Ricinus communis, H. brasiliensis, P. trichocarpa, Solanum lycopersicum, and O. sativa japonica species. (B) Heat map of expression patterns of S. glabra-specific NBS-LRR genes in various tissues.
