# Supplementary material for: The Diesel Tree Sindora glabra Genome Provides Insights Into the Evolution of Oleoresin Biosynthesis
Source: Front Plant Sci. 2022 Jan 4;12:794830. doi: 10.3389/fpls.2021.794830 (PMC8764381; doi:10.3389/fpls.2021.794830)
Supplement: Supplementary file 13 [file Data_Sheet_1.docx]

**Table S1** Statistics of Genome assembly.

| Sample ID | length | | number | |
| --- | --- | --- | --- | --- |
|  | Contig**(bp) | Scaffold(bp) | Contig** | Scaffold |
| Total | 1113810175 | 1,113,810,175 | 1,439 | 1439 |
| Max | 5,553,071 | 5,553,071 | - | - |
| Number>=2000 | - | - | 1,439 | 1439 |
| N50 | 1,297,087 | 1,297,087 | 264 | 264 |
| N60 | 1,067,416 | 1,067,416 | 359 | 359 |
| N70 | 870,960 | 870,960 | 473 | 473 |
| N80 | 682,226 | 682,226 | 617 | 617 |
| N90 | 469,620 | 469,620 | 812 | 812 |

**Table S2** Distribution of chromosome length.

| Chromosome | Chr_id | Length (bp) |
| --- | --- | --- |
| Hic_asm_0 | Chr.01 | 136,071,090 |
| Hic_asm_1 | Chr.02 | 116,316,258 |
| Hic_asm_2 | Chr.03 | 109,186,524 |
| Hic_asm_3 | Chr.04 | 91,914,064 |
| Hic_asm_4 | Chr.05 | 88,658,134 |
| Hic_asm_5 | Chr.06 | 84,872,623 |
| Hic_asm_6 | Chr.07 | 83,373,435 |
| Hic_asm_7 | Chr.08 | 81,990,953 |
| Hic_asm_8 | Chr.09 | 76,993,406 |
| Hic_asm_9 | Chr.10 | 75,912,673 |
| Hic_asm_10 | Chr.11 | 75,144,154 |
| Hic_asm_11 | Chr.12 | 71,117,942 |
| Total |  | 1,091,551,256 （98.0%） |

**Table S3** Statistics of final genome assembly.

| Sample ID | length | | number | |
| --- | --- | --- | --- | --- |
|  | Contig** (bp) | Scaffold (bp) | Contig**(bp) | Scaffold (bp) |
| Total | 111,,381,.0175 | 1,113,919,975 | 1,461 | 363 |
| Max | 5,205,380 | 136,071,090 | - | - |
| Number>=2000 | - | - | 1,461 | 363 |
| N50 | 1,273,373 | 84,872,623 | 267 | 6 |
| N60 | 1,043,588 | 83,373,435 | 363 | 7 |
| N70 | 856,073 | 81,990,953 | 480 | 8 |
| N80 | 668,303 | 75,912,673 | 626 | 10 |
| N90 | 466,750 | 75,144,154 | 824 | 11 |
|  | | | | |

**Table S4** BUSCO assessment.

| **Summary:** C: 90.9% [S: 81.5%, D: 9.4%], F: 1.8%, M: 7.3%, n: 1440 |  |
| --- | --- |
| Complete BUSCOs (C) | 1,309 |
| Complete and single-copy BUSCOs (S) | 1,174 |
| Complete and duplicated BUSCOs (D) | 136 |
| Fragmented BUSCOs (F) | 26 |
| Missing BUSCOs (M) | 105 |
| Total BUSCO groups searched | 1,440 |

**Table S5** Statistics of coverage rate.

| **% of Percentage** | | |
| --- | --- | --- |
| Reads  Genome | Mapping rate (%)  Average sequencing depth  Coverage (%)  Coverage at least 4X (%)  Coverage at least 10X (%)  Coverage at least 20X (%) | 94.14  32.55  97.64  95.86  92.47  82.18 |

**Table S6** Statistics of SNP calling.

| **Number** | **Percentage** |  |
| --- | --- | --- |
| All SNP  Heterozygosis SNP  Homology SNP | 5988177  5575137  413040 | 0.57%  0.53%  0.04% |

**Table S7** Statistic of Gene structure prediction.

|  | Gene set | Number | Average transcript length(bp) | Average CDS length(bp) | Average exons per gene | Average exon length(bp) | Average intron length(bp) |
| --- | --- | --- | --- | --- | --- | --- | --- |
| De novo | Augustus | 43,677 | 2,728.04 | 944.87 | 4.31 | 219.07 | 538.21 |
|  | GlimmerHMM | 84,616 | 11,704.62 | 542.97 | 3.36 | 161.45 | 4,723.19 |
|  | SNAP | 46,488 | 5,971.97 | 556.59 | 4.56 | 121.93 | 1,519.20 |
|  | Geneid | 65,471 | 5,989.79 | 630.52 | 3.82 | 165.09 | 1,900.98 |
|  | Genscan | 44,250 | 14,108.13 | 926.85 | 4.99 | 185.75 | 3,303.66 |
| Homolog | Car | 25,303 | 3,661.60 | 1,144.67 | 4.84 | 236.4 | 655.1 |
|  | Gma | 26,301 | 3,626.82 | 1,140.95 | 4.81 | 237.31 | 652.82 |
|  | Vun | 25,088 | 3,803.13 | 1,168.72 | 4.95 | 235.94 | 666.36 |
|  | Pvu | 26,650 | 3,516.06 | 1,118.83 | 4.72 | 237.23 | 645.09 |
|  | Ssu | 26,096 | 3,781.09 | 1,125.23 | 4.76 | 236.27 | 705.88 |
|  | Mtr | 25,225 | 3,625.28 | 1,143.27 | 4.86 | 235.33 | 643.3 |
| RNAseq | PASA | 242,686 | 3,724.76 | 952.75 | 5.09 | 187.17 | 677.69 |
|  | Transcripts | 54,715 | 9,670.85 | 2,355.80 | 6.91 | 340.76 | 1,237.05 |
| EVM | | 43,433 | 3,427.01 | 948.44 | 4.33 | 218.82 | 743.34 |
| Pasa-update* | | 43,165 | 3,506.76 | 954.66 | 4.32 | 220.9 | 768.3 |
| Final set* | | 31,944 | 4,239.44 | 1,143.30 | 5.02 | 227.58 | 769.45 |

**Table S8** Statistic of Gene structure among species.

| Species | Number | Average transcript length(bp) | Average CDS length(bp) | Average exons per gene | Average exon length(bp) | Average intron length(bp) |
| --- | --- | --- | --- | --- | --- | --- |
| Sindora_glabra | 31,944 | 4,239.44 | 1,143.30 | 5.02 | 227.58 | 769.45 |
| Mtr | 31,110 | 3,331.22 | 1,346.79 | 5.02 | 268.53 | 494.2 |
| Ssu | 31,106 | 4,127.42 | 1,104.33 | 4.91 | 225.1 | 773.97 |
| Pvu | 27,825 | 3,128.79 | 1,243.43 | 5.16 | 240.77 | 452.74 |
| Vun | 28,166 | 3,423.49 | 1,361.77 | 5.34 | 254.81 | 474.59 |
| Gma | 53,593 | 3,207.55 | 1,186.03 | 5.16 | 229.7 | 485.56 |
| Car | 24,399 | 3,583.85 | 1,293.82 | 5.45 | 237.56 | 515.06 |

**Table S9** Statistic of gene function annotation.

|  | Number | Percent(%) |
| --- | --- | --- |
| Total | 31,944 | - |
| Swissprot | 24,344 | 76.2 |
| Nr | 28,984 | 90.7 |
| KEGG | 23,660 | 74.1 |
| InterPro | 28,047 | 87.8 |
| GO | 17,141 | 53.7 |
| Pfam | 23,158 | 72.5 |
| Annotated | 29,184 | 91.4 |
| Unannotated | 2,760 | 8.6 |

**Table S10** Statistic of non-coding RNA annotation.

|  | | Copy number | Average length(bp) | Total length(bp) | % of genome |
| --- | --- | --- | --- | --- | --- |
| miRNA | | 701 | 132.81 | 93,098 | 0.008358 |
| tRNA | | 672 | 75.15 | 50,499 | 0.004533 |
| rRNA | rRNA | 87 | 251.2 | 21,854 | 0.001962 |
|  | 18S | 30 | 495.5 | 14,865 | 0.001334 |
|  | 28S | 11 | 135.27 | 1,488 | 0.000134 |
|  | 5.8S | 4 | 143.25 | 573 | 0.000051 |
|  | 5S | 42 | 117.33 | 4,928 | 0.000442 |
| snRNA | snRNA | 1,424 | 109.01 | 155,236 | 0.013936 |
|  | CD-box | 1,230 | 105.15 | 129,334 | 0.011611 |
|  | HACA-box | 55 | 129.65 | 7,131 | 0.00064 |
|  | splicing | 138 | 134.78 | 18,600 | 0.00167 |
|  | scaRNA | 1 | 171 | 171 | 0.000015 |
|  | Unknown | 0 | 0 | 0 | 0 |

**Table S11** Classification of repeat sequences.

|  | Denovo+Repbase |  | TE Proteins |  | Combined TEs |  |
| --- | --- | --- | --- | --- | --- | --- |
|  | Length(bp) | % in Genome | Length(bp) | % in Genome | Length(bp) | % in Genome |
| DNA | 53,978,212 | 4.85 | 5,369,216 | 0.48 | 55,710,989 | 5 |
| LINE | 10,135,756 | 0.91 | 2,210,841 | 0.2 | 11,595,221 | 1.04 |
| SINE | 24,220 | 0 | 0 | 0 | 24,220 | 0 |
| LTR | 362,429,670 | 32.54 | 82,009,245 | 7.36 | 373,539,494 | 33.53 |
| Unknown | 170,759,738 | 15.33 | 0 | 0 | 170,759,738 | 15.33 |
| Total | 567,210,504 | 50.92 | 89,588,728 | 8.04 | 571,621,404 | 51.32 |

**Table S12** Gene number used for gene family clustering.

| Symbol | ScientificName | geneNumber |
| --- | --- | --- |
| [Sgla](file:///F:\%25E7%2583%25AD%25E6%259E%2597%25E6%2589%2580\Experiments\%25E6%25B2%25B9%25E6%25A5%25A0\Genome%20sequencing\Results\Comparative%20genome\%25E6%2595%25B4%25E4%25BD%2593%25E7%2589%2588-Report-X101SC19071081-Z01\Report-X101SC19071081-Z01\src\html\-) | Sindora glabra | 31940 |
| [Gmax](ftp://ftp.ensemblgenomes.org/pub/plants/release-42/fasta/glycine_max/cds/Glycine_max.Glycine_max_v2.1.cds.all.fa.gz) | Glycine max | 55702 |
| [Vung](ftp://ftp.ncbi.nlm.nih.gov/genomes/all/GCF/004/118/075/GCF_004118075.1_ASM411807v1/GCF_004118075.1_ASM411807v1_genomic.gff.gz) | Vigna unguiculata | 28166 |
| [Pvul](ftp://ftp.ncbi.nlm.nih.gov/genomes/all/GCF/000/499/845/GCF_000499845.1_PhaVulg1_0/GCF_000499845.1_PhaVulg1_0_genomic.gff.gz) | Phaseolus vulgaris | 27825 |
| [Cari](ftp://ftp.ncbi.nlm.nih.gov/genomes/all/GCF/000/331/145/GCF_000331145.1_ASM33114v1/GCF_000331145.1_ASM33114v1_genomic.gff.gz) | Cicer arietinum | 24399 |
| [Mtru](ftp://ftp.ncbi.nlm.nih.gov/genomes/all/GCF/000/219/495/GCF_000219495.3_MedtrA17_4.0/GCF_000219495.3_MedtrA17_4.0_genomic.gff.gz) | Medicago truncatula | 31110 |
| [Cmic](https://www.ncbi.nlm.nih.gov/genome/57158?genome_assembly_id=405569) | Cinnamomum micranthum | 27854 |
| [Bpen](https://genomevolution.org/CoGe/GenomeInfo.pl?gid=35080) | Betula pendula | 24574 |
| [Egra](ftp://ftp.ncbi.nlm.nih.gov/genomes/refseq/plant/Eucalyptus_grandis/latest_assembly_versions/GCF_000612305.1_Egrandis1_0/GCF_000612305.1_Egrandis1_0_genomic.gff.gz) | Eucalyptus grandis | 35428 |
| [Oeur](https://phytozome.jgi.doe.gov/pz/portal.html#!info?alias=Org_Oeuropaea_er) | Olea europaea | 50373 |
| [Tcac](https://ftp.ncbi.nlm.nih.gov/genomes/Theobroma_cacao) | Theobroma cacao | 30640 |
| [Atha](ftp://ftp.ensemblgenomes.org/pub/plants/release-45/) | Arabidopsis thaliana | 27310 |
| [Ptri](ftp://ftp.ensemblgenomes.org/pub/plants/release-45/) | Populus trichocarpa | 41234 |
| [Hbra](https://www.ncbi.nlm.nih.gov/genome/503?genome_assembly_id=276723) | Hevea brasiliensis | 34648 |
| [Osat](ftp://ftp.ensemblgenomes.org/pub/plants/release-45/) | Oryza sativa | 35359 |
| [Ssub](https://ftp.ncbi.nlm.nih.gov/genomes/all/GCA/004/329/165/GCA_004329165.1_ASM432916v1/GCA_004329165.1_ASM432916v1_genomic.fna.gz) | Spatholobus suberectus | 31106 |
